# Effects of sodium bicarbonate, cholecalciferol, and protein supplementation interventions on muscle mass and metabolic disturbances in patients with chronic kidney disease: a systematic review and network meta-analysis

**DOI:** 10.3389/fnut.2026.1698991

**Published:** 2026-04-08

**Authors:** Shuilian Leng, Meijun Wu, Jingchun Yao, Xiaojuan Jiang

**Affiliations:** Department of Nephrology, Jiujiang University Affiliated Hospital, Jiujiang, Jiangxi Province, China

**Keywords:** cholecalciferol, chronic kidney disease, metabolic disturbances, muscle mass, network meta-analysis, protein supplementation, sodium bicarbonate

## Abstract

**Background:**

Patients with chronic kidney disease (CKD) often experience a decline in muscle mass and metabolic disturbances, which may increase the risk of cardiovascular events and all-cause mortality. Sodium bicarbonate, cholecalciferol, and protein supplementation are commonly used pharmacological and nutritional interventions; however, systematic evidence comparing their effects on muscle mass, metabolic status, and related outcomes in CKD patients remains lacking.

**Methods:**

We systematically searched PubMed, Embase, Web of Science, and the Cochrane Library from inception to July 1, 2025, and included eligible comparative clinical studies. Conventional meta-analysis and network meta-analysis (NMA) were used to compare the three categories of interventions in outcomes such as muscle mass, muscle function, and serum metabolic parameters, and surface under the cumulative ranking curve (SUCRA) values were used to rank intervention effects.

**Results:**

A total of 22 studies involving 2,879 patients were included, comprising 11 on sodium bicarbonate, 5 on cholecalciferol, and 6 on protein supplementation. Conventional meta-analysis indicated that sodium bicarbonate may be more effective in improving HCO₃^−^ and potassium levels in the early stage and may have certain effects on eGFR and systolic blood pressure at 24 months. NMA results showed that cholecalciferol was advantageous in increasing muscle mass (SMD = 0.68, 95% CI = 0.09 to 1.27), sodium bicarbonate performed better in improving serum albumin (SMD = 0.50, 95% CI = 0.01 to 0.99), and protein supplementation ranked highest for reducing serum phosphorus (SUCRA = 64.9%) and the incidence of adverse events (SUCRA = 71.9%). However, no significant differences were observed among the three interventions in muscle mass or serum metabolic parameters.

**Conclusion:**

Sodium bicarbonate and cholecalciferol may have potential advantages in improving serum albumin and increasing muscle mass, respectively. While protein supplementation may offer some value in reducing serum phosphorus and the incidence of adverse events. Given the limited number of included studies, small sample sizes, and substantial heterogeneity in intervention protocols, these conclusions should be further validated in future large-scale, rigorously designed randomized controlled trials.

**Systematic review registration:**

https://www.crd.york.ac.uk/PROSPERO/view/CRD420251126837, identifier PROSPERO (CRD420251126837).

## 1 Introduction

Chronic Kidney Disease (CKD) is a major global health issue, and alterations in patients’ nutritional status, body composition, and muscle mass are closely associated with its morbidity, mortality, and quality of life (1, 2). Recent epidemiological data indicate that kidney disease affects more than 850 million people worldwide, CKD affects over 10% of the global population, and CKD has become one of the leading causes of death globally, ranking ninth in 2023 (3–5). Notably, a decline in muscle mass or strength is not only a common comorbidity in CKD but has also been established as a manifestation of sarcopenia (6, 7), a condition characterized by the progressive loss of muscle strength, mass, or function (8). Among individuals with CKD, the prevalence of sarcopenia increases from approximately 10% in non–dialysis-dependent patients to 37% in those with End-Stage Kidney Disease (ESKD) (9, 10), and its presence is significantly associated with reduced quality of life, major adverse cardiovascular events, and all-cause mortality (7).

The etiology of muscle loss in patients with CKD is multifactorial, involving key mechanisms such as negative nitrogen balance, insufficient physical activity, sedentary behavior, metabolic acidosis (MA), and systemic inflammation (11, 12). Among these, MA is a common complication in advanced CKD, particularly when the glomerular filtration rate (GFR) falls below 30 mL/min/1.73 m^2^, with a prevalence of up to 30–50% (13). MA has been shown to exacerbate muscle catabolism through multiple pathways. Primarily, it serves as a potent stimulus for protein degradation, mainly by activating the ubiquitin–proteasome system (UPS) and caspase-3 pathways, and by promoting insulin resistance and growth hormone resistance (14, 15). In addition, MA can upregulate myostatin levels, inhibit myocyte growth, and impair the function of insulin-like growth factor-1 (IGF-1) (16, 17). Simultaneously, MA reduces albumin synthesis, leading to a persistent negative nitrogen balance (18). Moreover, MA interacts with bone metabolic abnormalities and is associated with lower bone mineral density; it promotes bone resorption, suppresses bone formation, and elevates parathyroid hormone (PTH) levels (19, 20). The interplay of sarcopenia, disordered bone metabolism, malnutrition, and acid–base imbalance results in a cascade of adverse changes in CKD patients, affecting muscle mass and function (e.g., sit-to-stand ability, body composition) as well as nutritional and mineral–bone metabolic parameters, and may further influence the progression of kidney dysfunction (21).

Therefore, exploring systematic intervention strategies based on pathophysiological mechanisms has become a key focus for clinicians, including alkali therapy to correct acidosis, treatments targeting mineral and bone metabolism, and nutritional support. For instance, CKD patients with serum HCO₃^−^ levels <22 mEq/L should receive sodium bicarbonate therapy (22). Previous small-scale clinical studies have suggested that correcting acidosis may improve muscle-related parameters (e.g., lower-limb function) and nutritional status (23, 24). However, serum bicarbonate levels >24 mEq/L may increase the risk of heart failure and mortality in CKD patients (25). Although a prior meta-analysis found that sodium bicarbonate supplementation did not affect handgrip strength or serum albumin levels, it lacked an evaluation of its effects on kidney function and blood pressure (26). Additionally, vitamin D deficiency is common in patients with advanced CKD. Oral cholecalciferol can effectively reduce parathyroid hormone (PTH) secretion and improve bone metabolism (27), and has become a standard treatment for secondary hyperparathyroidism in CKD. One previous study reported that vitamin D supplementation improved physical balance and function in CKD patients (28). However, the efficacy of cholecalciferol in improving muscle strength and serological metabolic parameters in this population remains uncertain.

Furthermore, given that inadequate protein intake is one of the key drivers of malnutrition in CKD, oral protein supplementation is considered an economical, feasible, and physiologically sound intervention strategy (29). Evidence indicates that, in malnourished dialysis patients, oral protein supplements can effectively promote positive nitrogen balance in both whole body and skeletal muscle, with effects comparable to intradialytic parenteral nutrition, and can confer sustained benefits even in the post-dialysis phase (30). Notably, whey protein, due to its high biological value and richness in branched-chain amino acids, shows advantages in stimulating protein uptake and synthesis to promote muscle recovery, making it a preferred choice in some studies (31). Taken together, protein supplementation holds potential for improving nutritional status and muscle-related phenotypes in CKD patients, though existing evidence remains heterogeneous, and systematic comparisons of its effects on muscle mass and multiple serological endpoints are still warranted.

However, to date, no network meta-analysis (NMA) has been conducted to systematically evaluate the potential effects of sodium bicarbonate, cholecalciferol, and protein supplementation on muscle mass, function, serum metabolic parameters, renal function, and blood pressure in patients. Therefore, the present NMA aims to investigate the impact of these pharmacological and protein supplementation interventions on sarcopenia, serum metabolism, and cardio-renal function–related outcomes in patients with CKD, thereby providing stronger evidence to guide clinical practice.

## 2 Methods

### 2.1 Protocol and registration

This study adhered to the Preferred Reporting Items for Systematic Reviews and Meta-Analyses (PRISMA) guidelines (32) and the associated extension for network meta-analyses (PRISMA-NMA) (33). The study protocol was prospectively registered in the PROSPERO database under registration number CRD420251126837.

### 2.2 Search strategy

We systematically searched PubMed, Embase, Web of Science, and the Cochrane Library from inception to July 1, 2025 for studies on interventions in patients with chronic kidney disease. Two reviewers independently performed the literature search, with discrepancies resolved through discussion with a third reviewer. We also screened the reference lists of relevant reviews to avoid missing eligible studies. The search strategy was developed using the following keywords: cholecalciferol, vitamin D3, sodium bicarbonate, baking soda, protein, protein supplement, amino acid, kidney disease, renal insufficiency, kidney failure, metabolic disturbance, muscle, muscle mass, muscle function, controlled clinical trial, randomized controlled trial. Detailed search strategies for the four databases are provided in Supplementary Table 1.

### 2.3 Eligibility criteria

The inclusion and exclusion criteria for this study followed the PICOS framework (Participants, Intervention, Comparison, Outcome, and Study). (1) Participants: Adult patients with a confirmed diagnosis of CKD, including both non–dialysis-dependent CKD and dialysis-dependent CKD. (2) Intervention: Oral administration of at least one of the sodium bicarbonate, cholecalciferol, or protein supplements (including amino acids or their derivatives). (3) Comparison: Control groups receiving placebo, usual care, or standard treatment. (4) Outcome: Primary outcomes included muscle mass, mid-arm muscle circumference (MAMC) and muscle function indicators, as well as serum metabolic parameters, including serum potassium, calcium, PTH, albumin, and bicarbonate levels. The incidence of adverse events (AEs) was also recorded. (5) Study: Randomized controlled trials (RCTs) and controlled trials published in English. Exclusion criteria: (1) Patients with severe comorbid conditions that could significantly affect muscle metabolism or nutritional status, such as active malignancy, severe liver disease, uncontrolled thyroid disorders, acute infection, or severe heart failure. (2) Interventions including other systemic medications or nutritional therapies, aside from the target supplements, that might significantly influence study outcomes. (3) Studies designed as non-controlled trials, reviews, case reports, animal experiments, or *in vitro* studies. (4) Studies with incomplete data or those from which required outcome measures could not be extracted. (5) Studies reporting only secondary outcomes or outcomes unrelated to the focus of this review.

### 2.4 Study selection and data extraction

Two reviewers independently conducted the study selection and data extraction processes, with any disagreements resolved through consultation with a third reviewer. During the study selection phase, duplicate records from the initially identified studies were removed using Endnote 20 (Clarivate Analytics, London, UK). Subsequently, titles and abstracts were screened to identify potentially relevant studies. Finally, the full texts of these studies were reviewed to determine eligibility according to the predefined criteria.

Additionally, two reviewers independently extracted relevant data using a standardized Excel spreadsheet, followed by cross-checking and verification. The extracted information included: first author, year of publication, country, study design, patient sample size, age, sex, body mass index (BMI), blood pressure, medical history, baseline laboratory results, route of intervention, frequency, and dosage. Continuous outcome measures included muscle mass, MAMC, sit-to-stand time, serum potassium, calcium, phosphorus, PTH, albumin, bicarbonate levels, renal function, and blood pressure. The dichotomous outcome measure was the incidence of adverse events (AEs). For graphical data, mean and standard deviation (SD) values were extracted using Origin 2024 (OriginLab, Massachusetts, USA). Finally, missing data were addressed by attempting to contact the study authors.

### 2.5 Risk of bias assessment

As the included studies comprised both RCTs and cohort studies, we applied the Cochrane Risk of Bias (RoB) tool (34) and the Newcastle–Ottawa Scale (NOS) (35) for quality assessment, respectively. For RCTs, the RoB tool evaluates seven domains: random sequence generation, allocation concealment, blinding of participants and personnel, blinding of outcome assessors, incomplete outcome data, selective outcome reporting, and other potential sources of bias. Each domain is rated as having a “low risk,” “high risk,” or “unclear risk” of bias. For cohort studies, the NOS assesses study quality across three domains: selection of study groups, comparability between groups, and outcome assessment, comprising a total of eight items. Studies are scored using a star system (maximum of nine stars), with a higher score indicating better quality. Two reviewers performed independent assessments, and any disagreements were resolved through discussion or arbitration with a third reviewer. The final quality assessment results will be presented in both graphical and tabular formats in the Results section.

### 2.6 Statistical analysis

We performed conventional meta-analyses using Review Manager (version 5.4, The Cochrane Collaboration), calculating effect sizes based on direct evidence. For categorical data, such as the incidence of adverse events, odds ratios (ORs) with 95% confidence intervals (CIs) were used. For continuous data, including muscle mass, muscle function, and serum metabolic parameters, standardized mean differences (SMDs) with corresponding 95% CIs were calculated. Statistical heterogeneity was assessed using the *I*^2^ statistic, with *I*^2^ > 50% indicating substantial heterogeneity and *I*^2^ ≥ 75% indicating considerable heterogeneity. Sensitivity analyses were conducted to evaluate the robustness of pooled results when at least five studies were included in the analysis. Subgroup analysis was conducted to identify potential factors influencing heterogeneity.

NMA was conducted using the “mvmeta” and “Network” packages in Stata/SE (version 16.0) (36). The analysis compared differences among various interventions in terms of muscle mass, serum metabolic parameters, and incidence of adverse events. A network evidence plot was generated to visually display the strength of evidence between interventions, where node size represented sample size and the thickness of connecting lines indicated the number of direct comparison studies. Surface under the cumulative ranking curve (SUCRA) values were used to rank the interventions, with scores closer to 100% indicating a higher probability of being the most effective. Publication bias was assessed using funnel plots. A *p*-value < 0.05 was considered statistically significant.

## 3 Results

### 3.1 Literature selection

**Figure 1:**
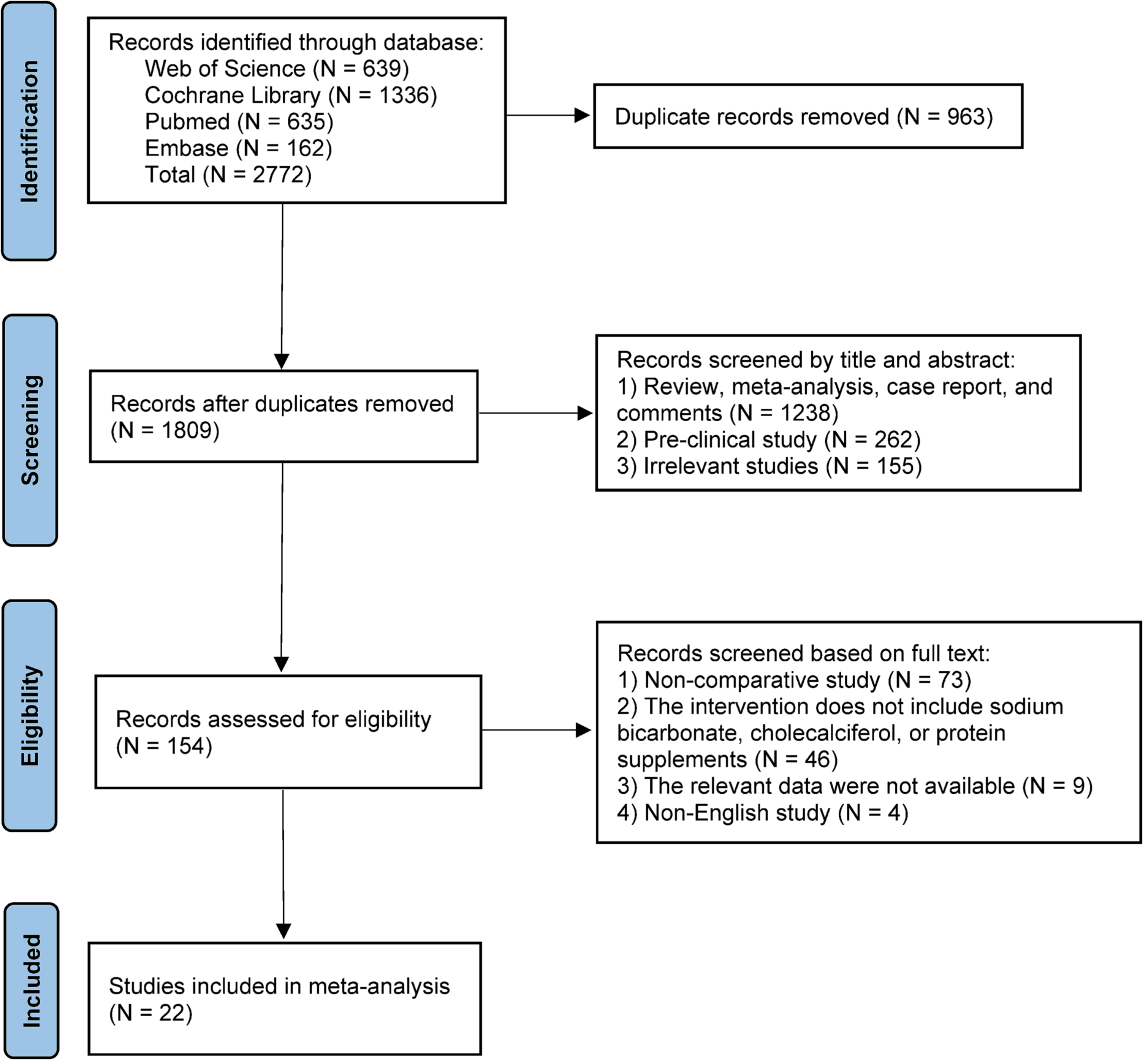
PRISMA flowchart of the study selection process.

A total of 2,772 records were retrieved from the primary databases. After removing duplicates (*N* = 963), 1,809 records remained for title and abstract screening. Subsequently, 154 articles underwent full-text review, and 22 studies meeting the eligibility criteria were ultimately included in the meta-analysis. The study selection process is illustrated in Figure 1.

### 3.2 Characteristics of included trials

A total of 22 studies (37–58) (23 datasets) were included, comprising 11 on sodium bicarbonate, 5 on cholecalciferol, and 6 on protein supplementation, all published in English. Nineteen were randomized controlled trials (RCTs) and three were cohort studies, with publication years ranging from 2005 to 2025. Sixteen studies reported changes in muscle mass following pharmacological or protein supplementation interventions. Muscle mass in the included trials was assessed using a variety of methods, including Bioelectrical Impedance Analysis (BIA), Bioelectrical Impedance Spectroscopy (BIS), and mid-arm muscle circumference (AMC). 15 studies reported changes in serum metabolic parameters, and 18 studies reported the incidence of adverse events.

Regarding baseline patient characteristics, the 22 included studies involved a total of 2,879 participants, comprising 1,813 males and 1,066 females. Of these, 1,474 were assigned to intervention groups and 1,405 to control groups. Participant ages ranged from 20.5 to 82.8 years. Sixteen studies reported BMI, 13 reported blood pressure, and 8 reported concomitant medication use. For intervention characteristics, all pharmacological or protein supplementation interventions were administered orally. Dosages varied across studies: sodium bicarbonate ranged from 0.3 to 1.2 mEq/kg, cholecalciferol from 20,000 to 50,000 U, and protein supplements from 15 to 30 g. The frequency of sodium bicarbonate administration was three times daily in six studies and twice daily in one study. Cholecalciferol was administered weekly in three studies and once daily in one study. Protein supplementation was provided three times per week (*N* = 3), twice daily (*N* = 2), or once daily (*N* = 1). The main characteristics of the different pharmacological and protein supplementation interventions are summarized in Supplementary Tables 1–3.

### 3.3 Risk of bias assessment

**Figure 2:**
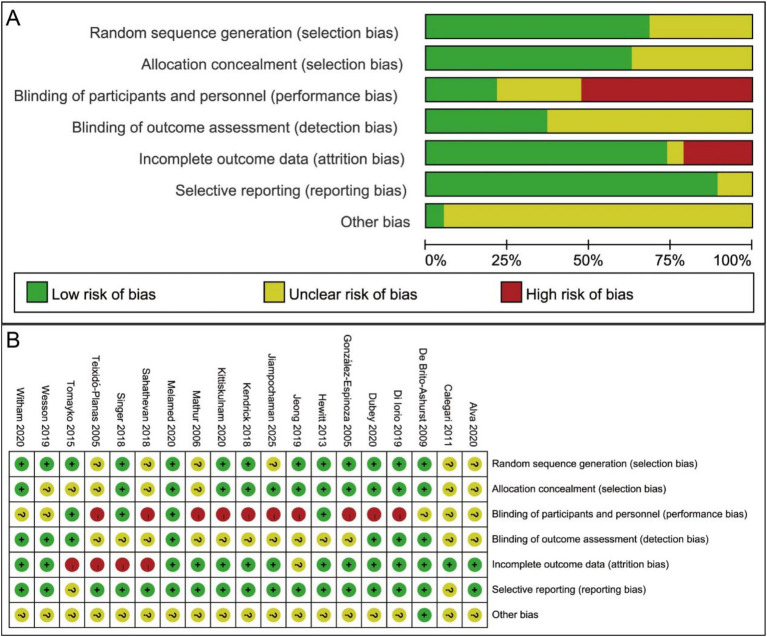
The risk of bias graph for included RCTs **(A)** Risk of bias graph; **(B)** Risk of bias summary. RCTs: randomized controlled trials.

The potential risk of bias for the 19 RCTs was assessed using the RoB tool, with the results presented in Figures 2A,B. None of the studies were rated as having a low risk of bias across all seven domains; all contained a mix of unclear and low risk assessments. Specifically, 13 studies reported the method of randomization, and 12 clearly described allocation concealment. Regarding blinding, 10 studies were explicitly “open-label” and were considered at high risk of performance bias. Seven studies were judged to have a low risk of detection bias. Four studies were considered at high risk of attrition bias. Seventeen studies were rated as having a low risk of reporting bias. For other bias, only one study was judged to have a low risk, with balanced baseline characteristics among participants. Overall, the included RCTs were predominantly of low risk of bias, indicating acceptable quality. Additionally, the methodological quality of the cohort studies was evaluated using the NOS, as shown in Supplementary Table 2. Two cohort studies received a score of 8, and one study scored 8.5, all of which were classified as high-quality studies.

### 3.4 Results of conventional meta-analysis

#### 3.4.1 Sodium bicarbonate vs. control

**Figure 3:**
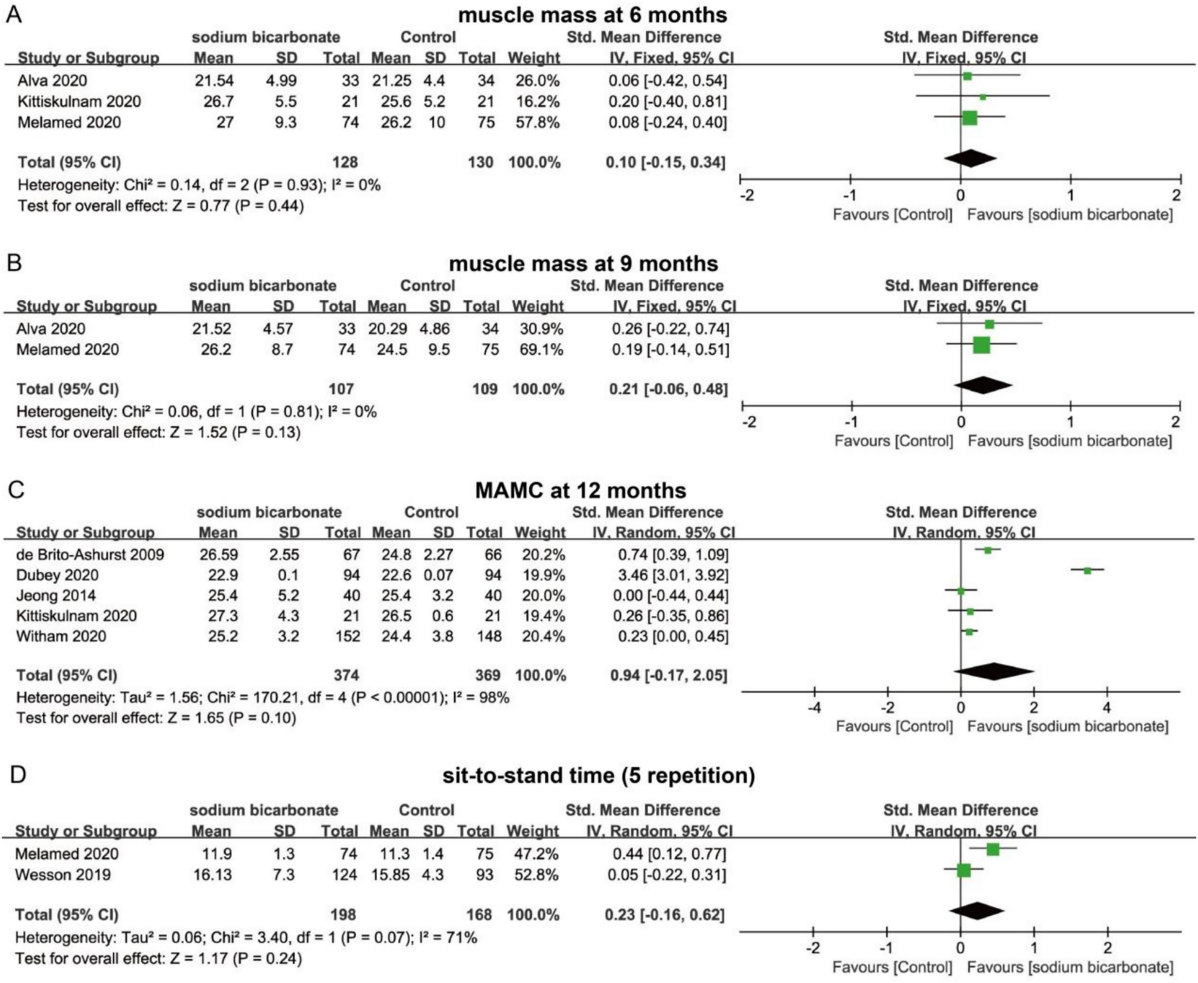
Forest plots illustrating the effect of sodium bicarbonate vs. control on muscle mass and function at different time points. **(A)** muscle mass at 6 months. **(B)** muscle mass at 9 months. **(C)** MAMC at 12 months. **(D)** sit-to-stand time (5 repetition).

Sodium bicarbonate is primarily used in the treatment of chronic kidney disease to correct metabolic acidosis, slow the progression of renal function decline, and alleviate related symptoms. In this review, we focused on changes in muscle mass, muscle function, serum metabolic parameters, renal function, and blood pressure following sodium bicarbonate intervention, and synthesized the findings through conventional meta-analysis. To evaluate the sustained effects of sodium bicarbonate on muscle mass in CKD patients, we performed pooled analyses for different follow-up time points. The results indicated that, compared with the control group, sodium bicarbonate did not produce a statistically significant improvement in muscle mass at 3, 6, or 9 months (*p* > 0.05) (Figures 3A–C).

Five studies, involving 743 patients, reported MAMC at 12 months. Pooled analysis showed that sodium bicarbonate intervention did not result in a significant increase in MAMC after 12 months (SMD = 0.94, 95% CI = −0.17 to 2.05, *p* > 0.05), with substantial heterogeneity observed (*I*^2^ = 98%) (Figure 3D). In addition, two studies reported sit-to-stand time (five repetitions) in CKD patients following sodium bicarbonate treatment. The pooled analysis indicated no significant reduction in sit-to-stand time compared with the control group (SMD = 0.23, 95% CI = −0.16 to 0.62, *p* > 0.05) (Figure 3E). Current evidence suggests that sodium bicarbonate intervention may not promote improvements in muscle mass or function in patients with CKD.

**Figure 4:**
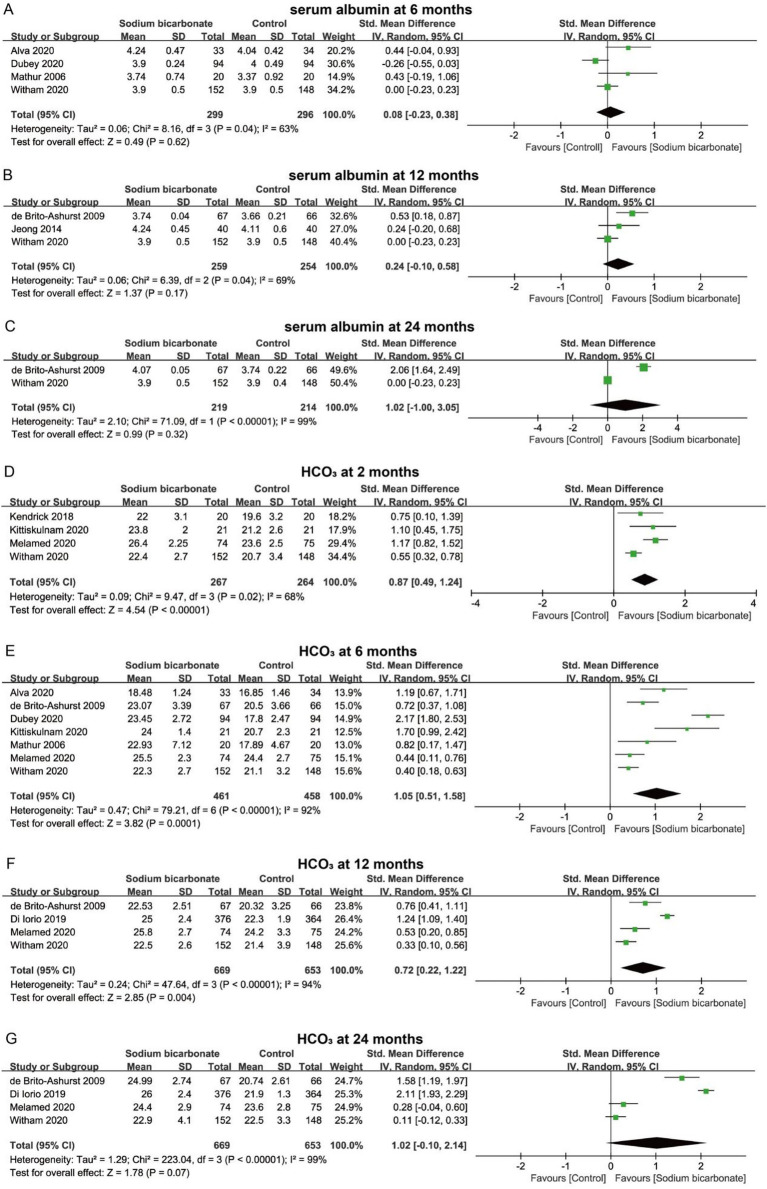
Forest plots showing the effect of sodium bicarbonate vs. control on serum albumin and HCO_3_^−^ at different time points. **(A)** Serum albumin at 6 months; **(B)** serum albumin at 12 months; **(C)** serum albumin at 24 months; **(D)** serum HCO_3_^−^ at 2 months; **(E)** serum HCO_3_^−^ at 6 months; **(F)** serum HCO_3_^−^ at 12 months; **(G)** serum HCO_3_^−^ at 24 months.

**Figure 5:**
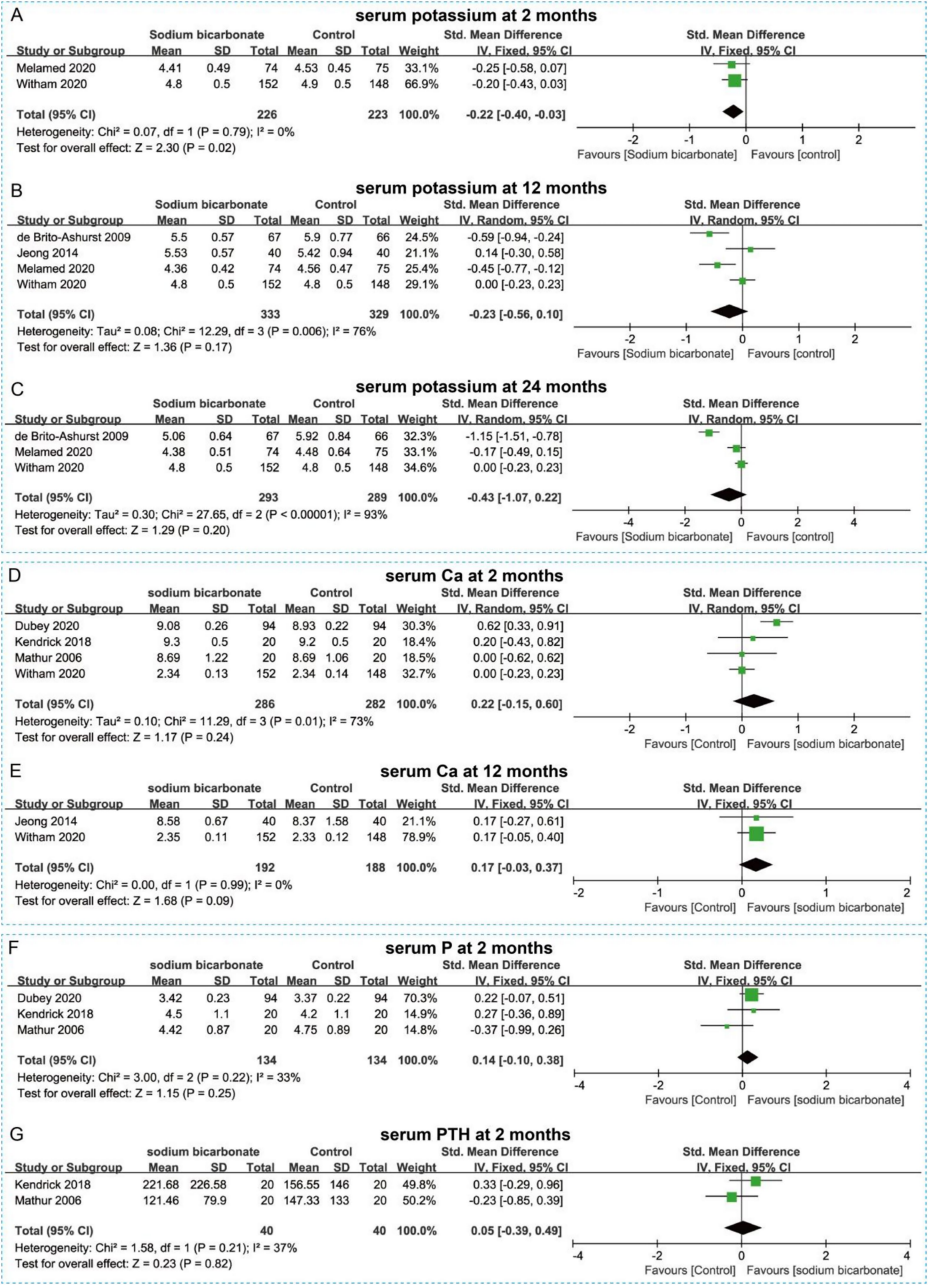
Forest plots illustrating the effect of sodium bicarbonate vs. control on serum metabolic parameters at different time points. **(A)** Serum potassium at 2 months. **(B)** Serum potassium at 12 months. **(C)** Serum potassium at 24 months. **(D)** Serum Ca at 2 months. **(E)** Serum Ca at 12 months. **(F)** Serum P at 2 months. **(G)** Serum PTH at 2 months.

Patients with CKD often present with varying degrees of protein and electrolyte imbalances; therefore, we further analyzed changes in serum metabolic parameters. For serum albumin levels, pooled analyses showed that sodium bicarbonate did not increase serum albumin at 6, 12, or 24 months compared with the control group (*p* > 0.05) (Figures 4A–C). Metabolic acidosis is a common complication of CKD, and serum HCO₃^−^ concentration is an important marker for its correction. The pooled results indicated that sodium bicarbonate significantly increased serum HCO₃^−^ levels at 2 months (SMD = 0.87, 95% CI = 0.49 to 1.24, *p* < 0.00001), 6 months (SMD = 1.05, 95% CI = 0.51 to 1.58, *p* = 0.0001), and 12 months (SMD = 0.72, 95% CI = 0.22 to 1.22, *p* = 0.004), but the change was not significant at 24 months (SMD = 1.02, 95% CI = −0.10 to 2.14, *p* > 0.05) (Figures 4D–G). For serum potassium, sodium bicarbonate significantly reduced levels at 2 months (SMD = −0.22, 95% CI = −0.40 to −0.03, *p* = 0.02), but no significant improvements were observed at 12 or 24 months (*p* > 0.05) (Figures 5A–C). In addition, serum calcium, phosphorus, and PTH levels were analyzed to assess the regulatory effects of sodium bicarbonate on bone metabolism in CKD patients. The pooled results showed no significant changes in serum calcium at 2 or 12 months (*p* > 0.05) (Figures 5D,E), and no significant changes in serum phosphorus or PTH at 2 months (*p* > 0.05) (Figures 5F,G).

**Figure 6:**
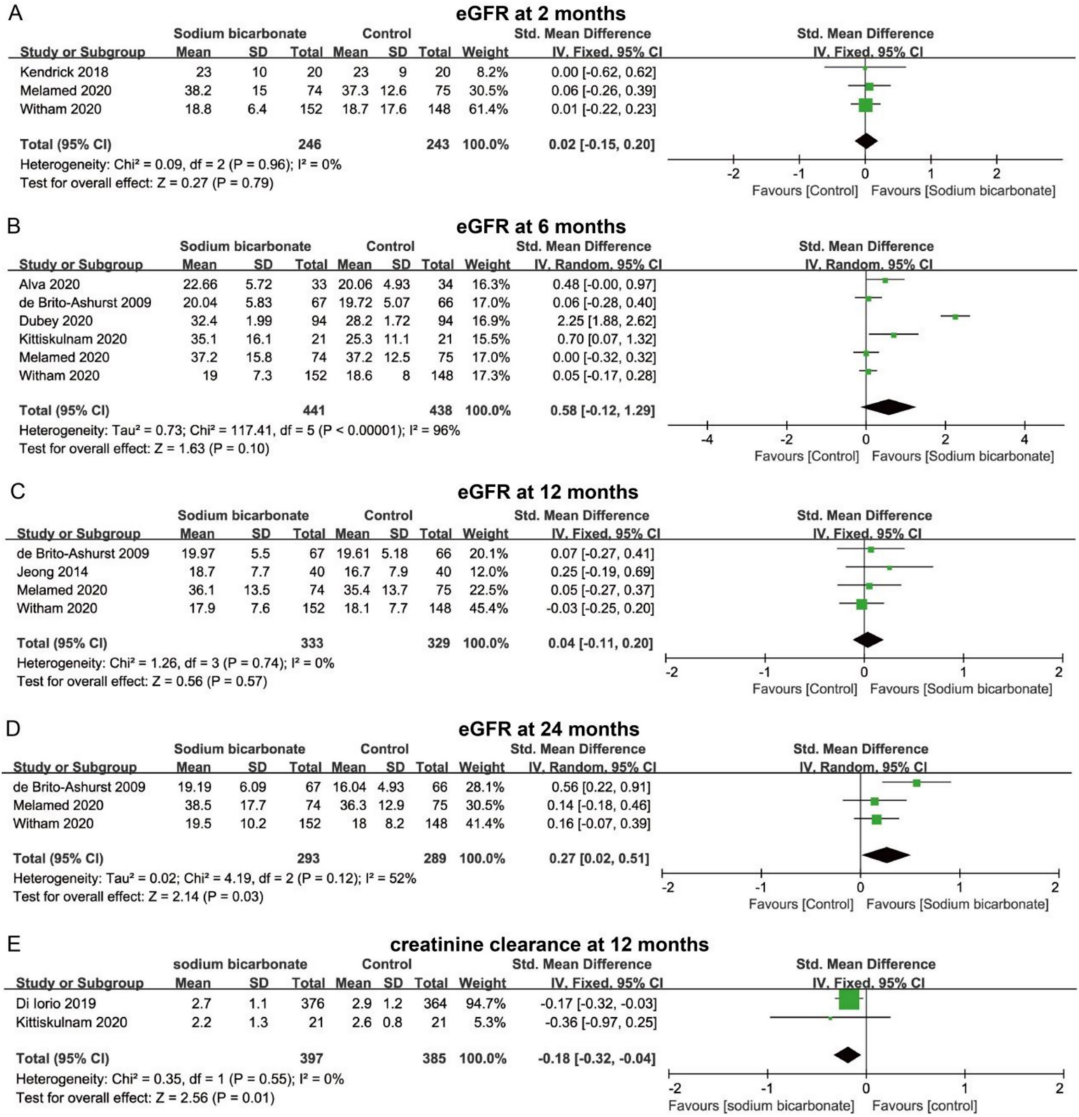
Forest plots showing the effect of sodium bicarbonate vs. control on renal function at different time points: **(A)** eGFR at 2 months, **(B)** eGFR at 6 months, **(C)** eGFR at 12 months, **(D)** eGFR at 24 months, **(E)** Creatinine clearance at 12 months.

**Figure 7:**
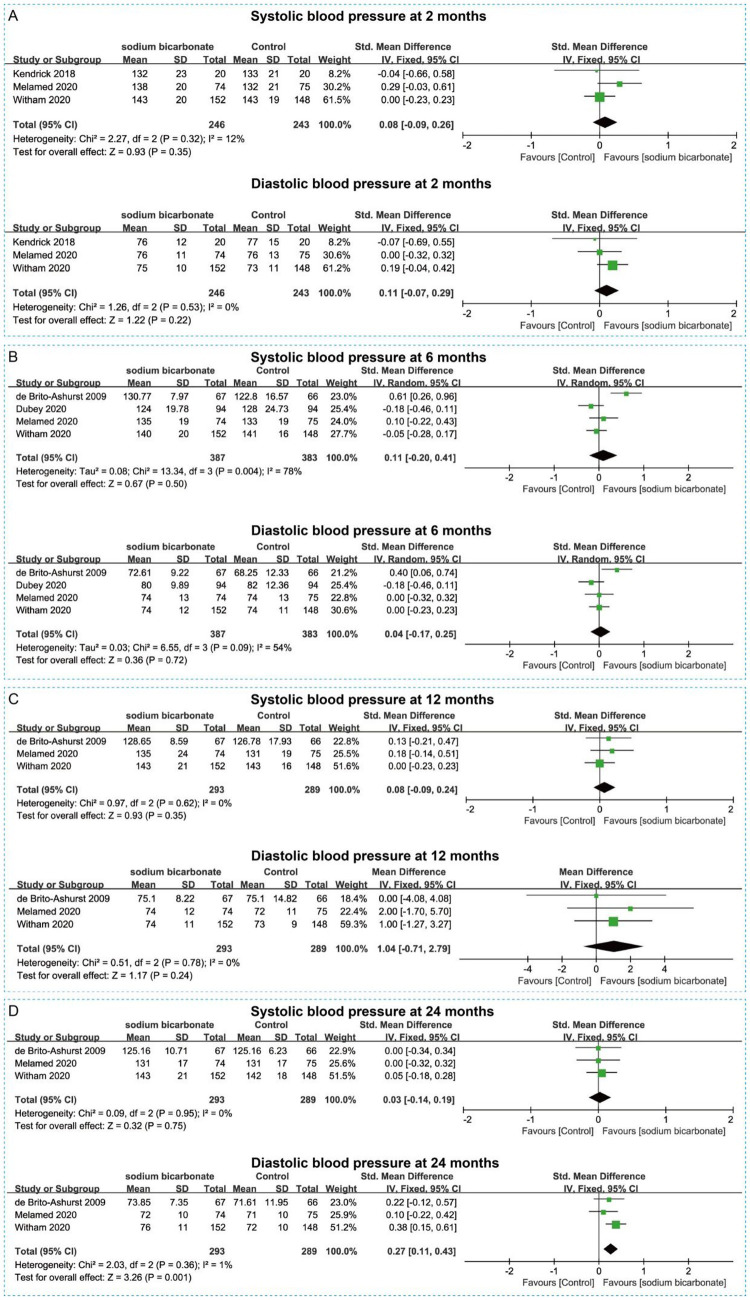
Forest plots illustrating the effect of sodium bicarbonate vs. control on blood pressure at different time points. **(A)** Systolic and diastolic blood pressure at 2 months. **(B)** Systolic and diastolic blood pressure at 6 months. **(C)** Systolic and diastolic blood pressure at 12 months. **(D)** Systolic and diastolic blood pressure at 24 months.

We also analyzed changes in renal function and blood pressure between the two groups. Pooled results indicated that sodium bicarbonate did not significantly improve estimated glomerular filtration rate (eGFR) at 2, 6, or 12 months (*p* > 0.05) (Figures 6A–C). However, compared with the control group, the sodium bicarbonate group showed a significant increase in eGFR at 24 months (SMD = 0.27, 95% CI = 0.02 to 0.51, *p* = 0.03) (Figure 6D). Additionally, sodium bicarbonate significantly reduced creatinine clearance at 12 months (SMD = −0.18, 95% CI = −0.32 to −0.04, *p* > 0.05) (Figure 6E). For systolic and diastolic blood pressure, no significant differences were observed between the two groups at 2, 6, or 12 months (*p* > 0.05) (Figures 7A–C). At 24 months, there was still no significant difference in diastolic blood pressure; however, sodium bicarbonate significantly increased systolic blood pressure at 24 months (SMD = 0.27, 95% CI = 0.11 to 0.43, *p* = 0.001) (Figure 7D).

Additionally, given the significant heterogeneity in the summary analysis results of serum HCO₃^−^ levels at 6 months, MAMC at 12 months, and eGFR at 6 months, subgroup analyses were conducted to explore potential sources of heterogeneity (Supplementary Figures 1–9). The subgroup analysis results based on different CKD stages, intervention frequencies, and intervention doses show that the CKD stage 3–4 subgroup, CKD stage 4–5 subgroup, dose ≤ 0.3 mEq/kg subgroup, dose > 0.3 mEq/kg subgroup, and three times a day subgroup all improved serum HCO₃^−^ levels. The dose ≤ 0.3 mEq/kg subgroup improved MAMC at 12 months, while no significant differences in eGFR at 6 months were observed across the subgroups. However, the limited number of studies included in each subgroup, the small sample size of CKD patients, and the significant heterogeneity within each subgroup warrant caution in further interpreting these results. Sex-based subgroup analyses were not performed because sex-stratified outcome data were not consistently available across the included studies.

In summary, sodium bicarbonate intervention may exert beneficial effects on early serum HCO₃^−^ and potassium levels, as well as on renal function and blood pressure at 24 months, while showing no significant improvement in muscle mass, muscle function, or serum calcium, phosphorus, PTH, and albumin levels. However, given the limited number of studies included and the relatively small sample sizes, these findings should be interpreted with caution.

#### 3.4.2 Cholecalciferol vs. control

**Figure 8:**
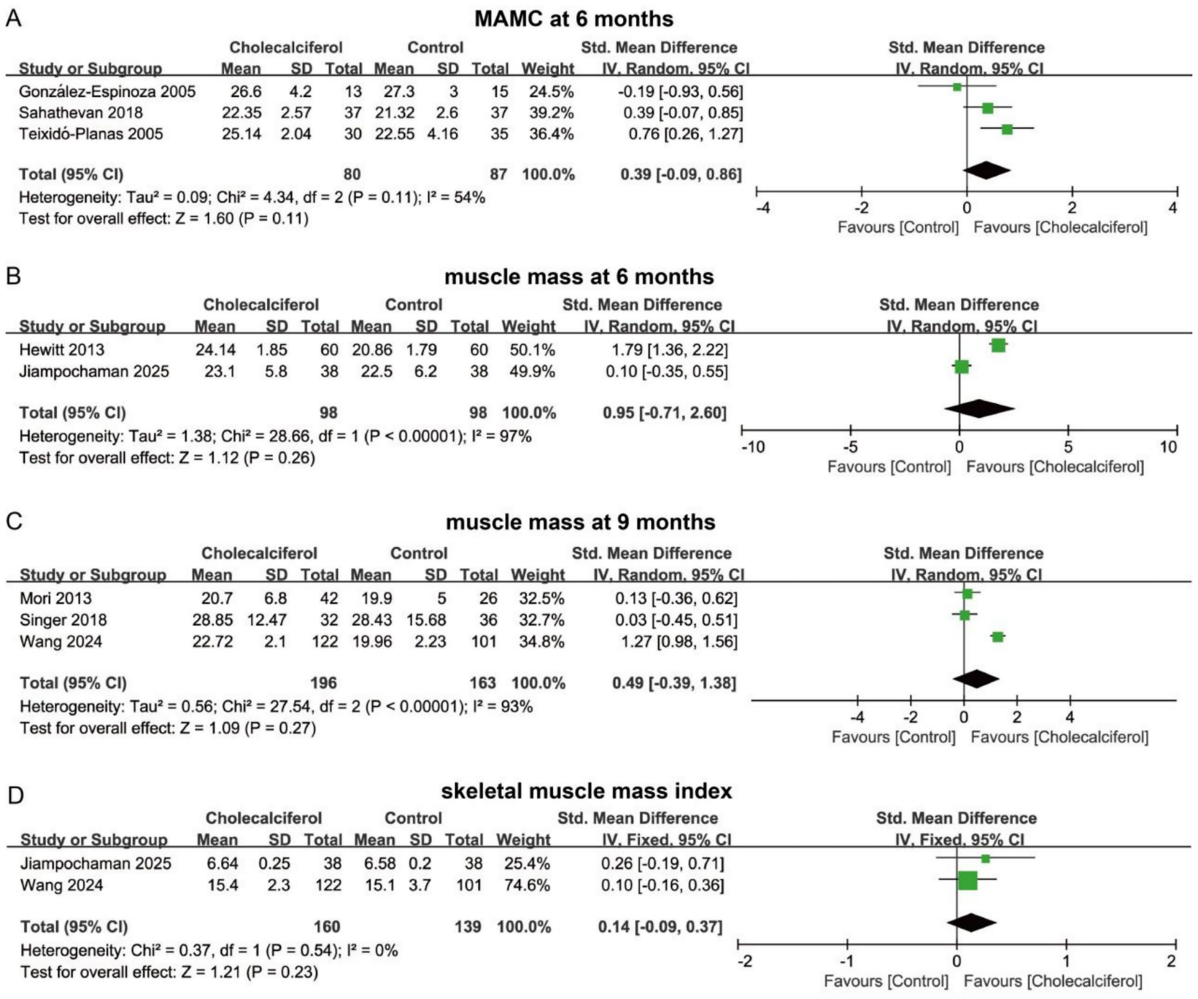
Forest plots illustrating the effect of cholecalciferol vs. control on muscle mass at different time points: **(A)** MAMC at 6 months; **(B)** muscle mass at 6 months; **(C)** muscle mass at 9 months; **(D)** skeletal muscle mass index.

**Figure 9:**
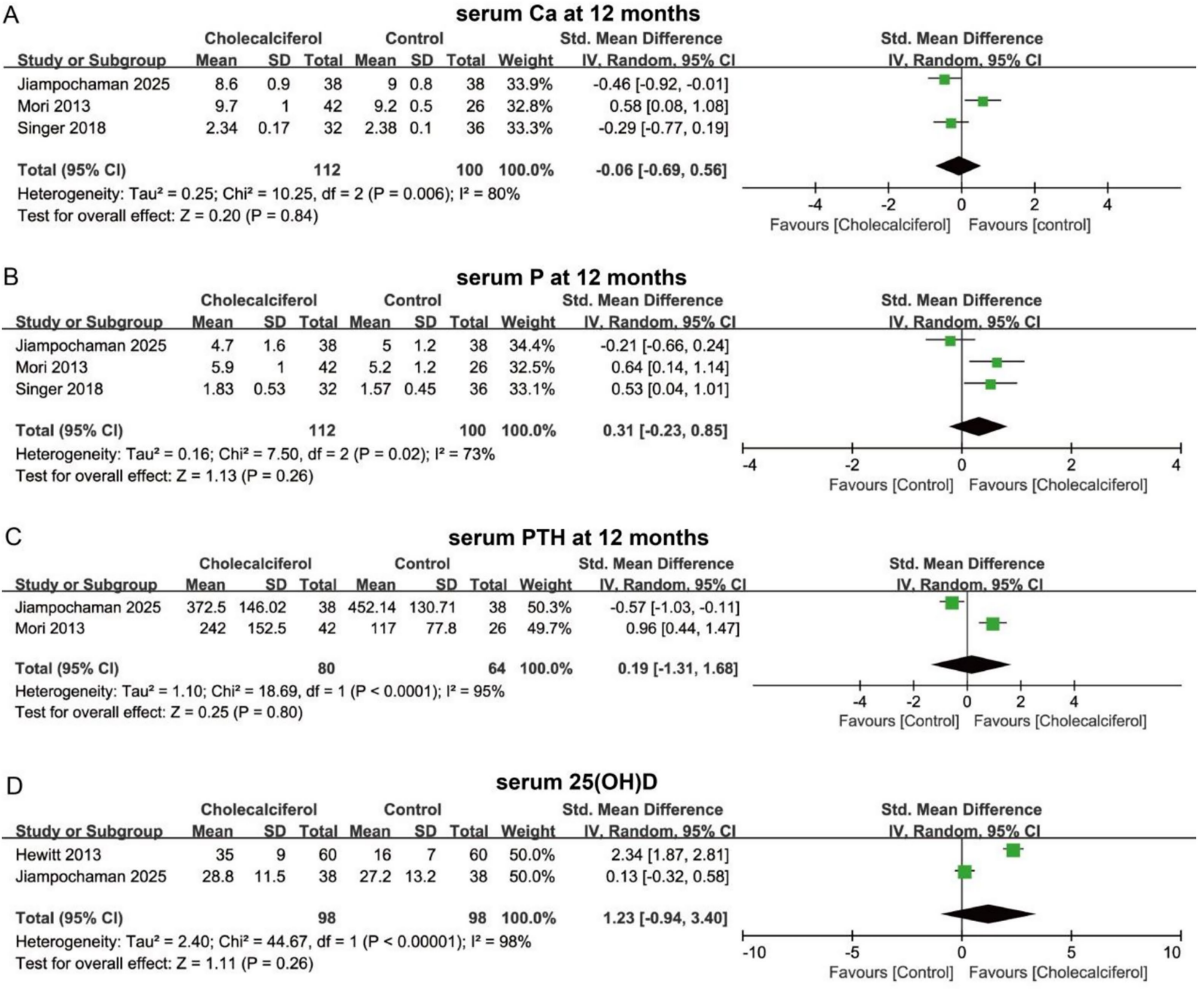
Forest plots showing the effect of cholecalciferol vs. control on serum metabolic parameters at different time points. **(A)** Serum Ca at 12 months. **(B)** Serum P at 12 months. **(C)** Serum PTH at 12 months. **(D)** Serum 25(OH)D.

In terms of muscle mass and function, cholecalciferol intervention did not significantly increase MAMC at 6 months (SMD = 0.39, 95% CI = −0.09 to 0.86, *p* > 0.05) (Figure 8A). No significant differences in muscle mass were observed between groups at 6 months (SMD = 0.95, 95% CI = −0.71 to 2.60, *p* > 0.05) or 9 months (SMD = 0.49, 95% CI = −0.39 to 1.38, *p* > 0.05) (Figures 8B,C). In addition, cholecalciferol did not increase skeletal muscle mass index (SMD = 0.14, 95% CI = −0.09 to 0.37, *p* > 0.05) (Figure 8D). For biochemical parameters, cholecalciferol intervention did not significantly improve serum calcium, serum phosphorus, or serum PTH levels at 12 months in CKD patients (*p* > 0.05). Likewise, serum 25(OH)D levels showed no significant difference between groups (SMD = 1.23, 95% CI = −0.94 to 3.40, *p* > 0.05) (Figure 9D). In summary, cholecalciferol intervention may not significantly improve muscle mass, muscle function, or metabolic disturbances in patients with CKD.

#### 3.4.3 Protein supplementation vs. control

**Figure 10:**
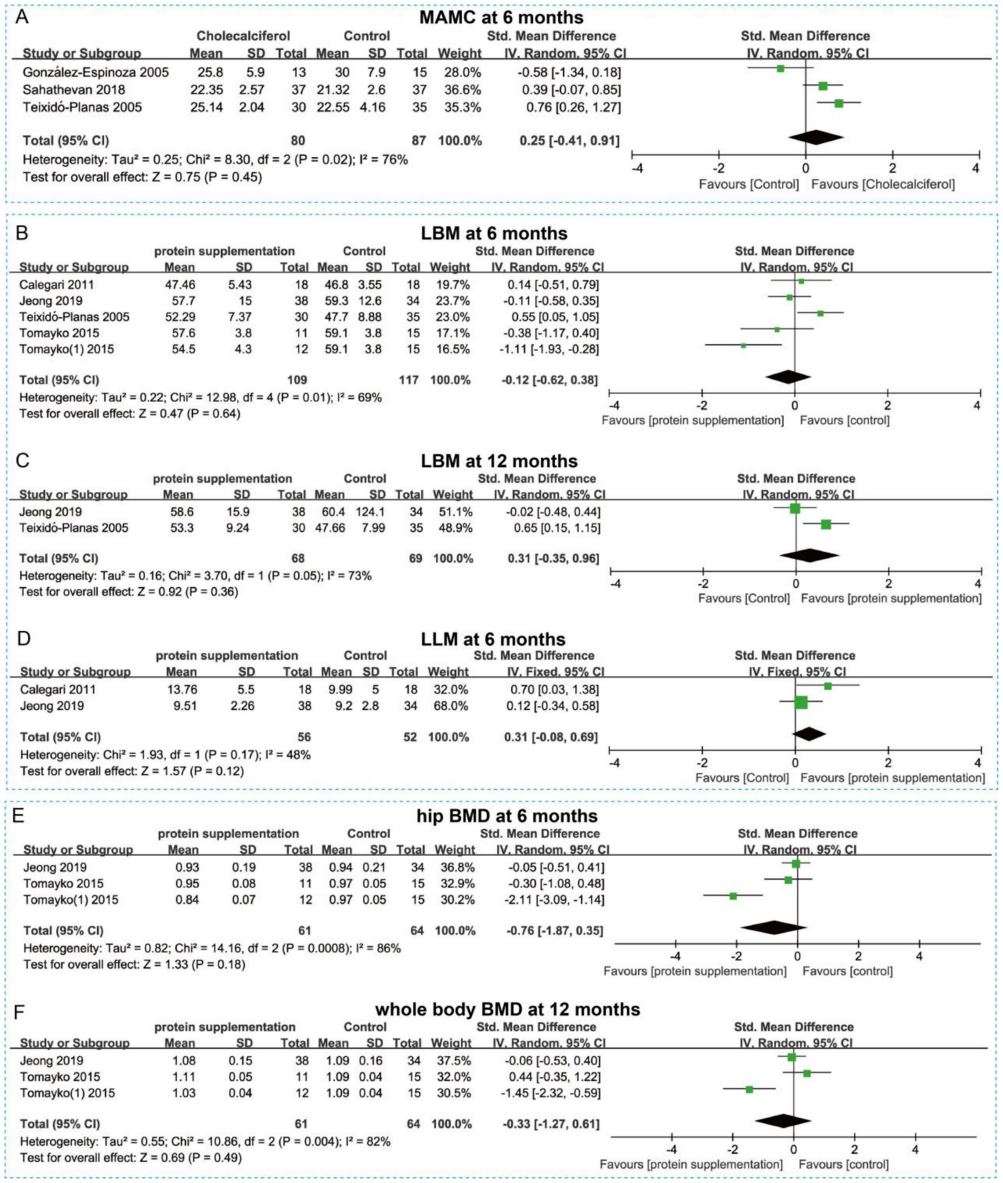
Forest plots illustrating the effect of protein supplementation vs. control on muscle mass and BMD at different time points. **(A)** MAMC at 6 months. **(B)** LBM at 6 months. **(C)** LBM at 12 months. **(D)** LLM at 6 months. **(E)** Hip BMD at 6 months. **(F)** Whole body BMD at 12 months.

**Figure 11:**
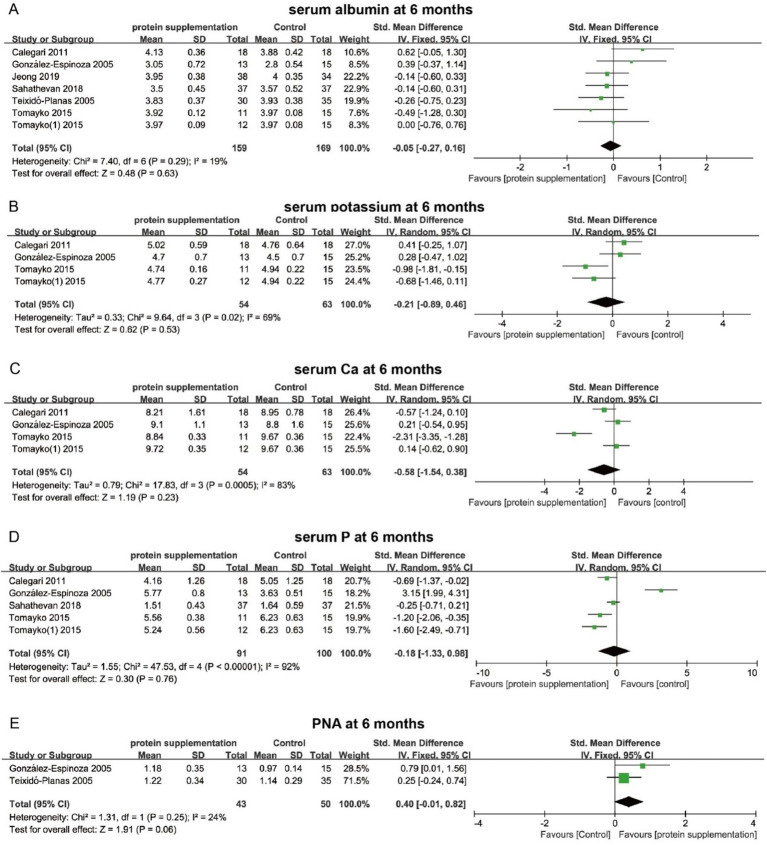
Forest plots showing the effect of protein supplementation vs. control on serum metabolic parameters at different time points. **(A)** Serum albumin at 6 months. **(B)** Serum potassium at 6 months. **(C)** Serum Ca at 6 months. **(D)** Serum P at 6 months. **(E)** PNA at 6 months.

Six studies on protein supplementation reported data on muscle mass, muscle function, and biochemical parameters. For muscle mass and function, pooled analysis showed that protein supplementation did not significantly increase MAMC at 6 months (SMD = 0.25, 95% CI = −0.41 to 0.91, *p* > 0.05) (Figure 10A). No significant differences were observed between groups for lean body mass (LBM) at either 6 months or 12 months (*p* > 0.05) (Figures 10B,C). Similarly, leg lean mass (LLM) at 12 months showed no significant difference between groups (SMD = 0.31, 95% CI = −0.08 to 0.69, *p* > 0.05) (Figure 10D). Additionally, protein supplementation did not significantly improve hip bone mineral density (BMD) at 6 months (SMD = −0.76, 95% CI = −1.87 to 0.35, *p* > 0.05) (Figure 10E) or whole-body BMD at 12 months (SMD = −0.33, 95% CI = −1.27 to 0.61, *p* > 0.05) (Figure 10F). For biochemical parameters, protein supplementation showed no significant effects on serum albumin, potassium, calcium, phosphorus, or protein nitrogen appearance rate (PNA) (Figures 11A–E). In summary, protein supplementation may not exert a beneficial effect on improving muscle mass, muscle function, or metabolic disturbances in patients with CKD.

Additionally, no significant effects of different doses and administration frequencies of protein supplements on LBM and serum albumin at 6 months were observed through subgroup analysis (Supplementary Figures 10–15). Although the three times a day subgroup reduced serum P levels, the limited number of included studies restricts the interpretability of this result.

### 3.5 Network meta-analysis of muscle mass

**Figure 12:**
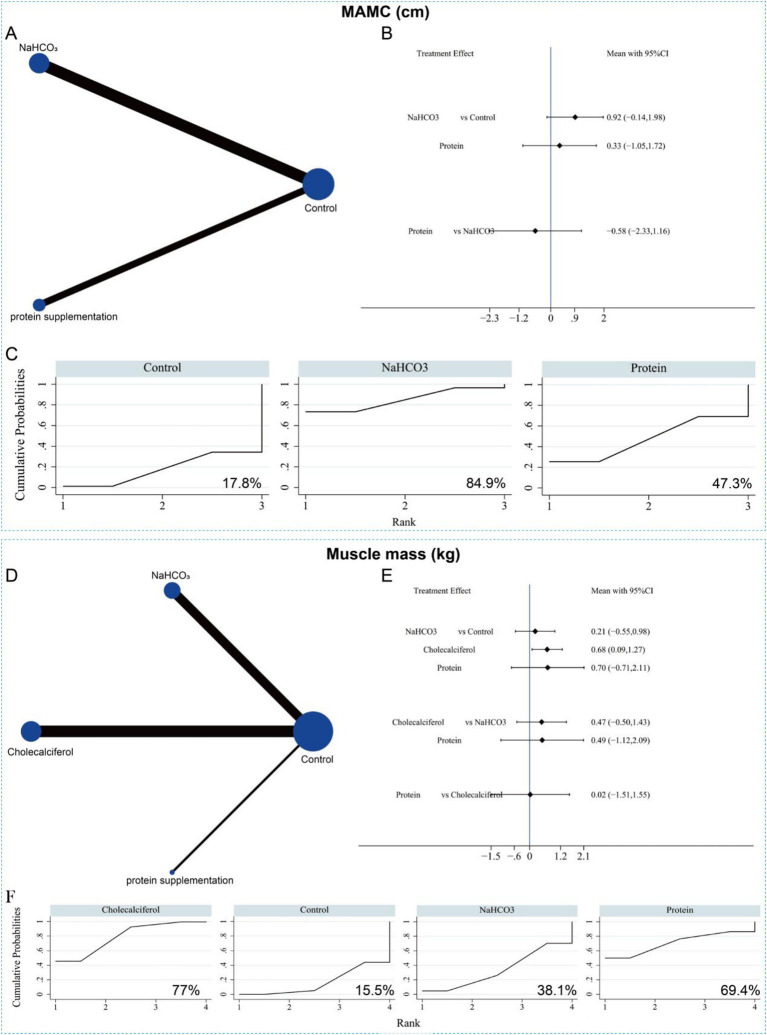
Network meta-analysis of the different interventions on MAMC. **(A)** Evidence network diagram of MAMC. **(B)** Forest plot showing comparative evidence for sodium bicarbonate and protein supplementation. **(C)** SUCRA curve and area under the curve (%) for MAMC after different interventions. **(D)** Evidence network diagram of muscle mass. **(E)** Forest plot showing comparative evidence for three different interventions. **(F)** SUCRA curve and area under the curve (%) for muscle mass after three different interventions.

To compare the effects of sodium bicarbonate, cholecalciferol, and protein supplementation on muscle mass and function in patients with CKD, an NMA was conducted focusing on key indicators, including MAMC and muscle mass. Eight studies, involving 910 CKD patients, reported MAMC outcomes for two interventions (sodium bicarbonate and protein supplementation). The network evidence plot is shown in Figure 12A. Compared with the control group, neither sodium bicarbonate nor protein supplementation significantly increased MAMC, which is consistent with the findings from the conventional meta-analysis (Figure 12B). Furthermore, no significant difference was observed between the two interventions in terms of MAMC (SMD = −0.58, 95% CI = −2.33 to 1.16). Based on SUCRA rankings, sodium bicarbonate had the highest probability of being the most effective intervention for increasing MAMC (*p* = 84.9%), followed by protein supplementation (*p* = 47.3%) (Figure 12C and Supplementary Table 3).

Nine studies, involving a total of 849 patients, reported muscle mass outcomes for the three interventions. The network evidence plot for these comparisons is shown in Figure 12D. Compared with the control group, cholecalciferol significantly increased muscle mass in patients with CKD (SMD = 0.68, 95% CI = 0.09 to 1.27) (Figure 12E). In contrast, sodium bicarbonate and protein supplementation did not show significant improvements in muscle mass. No significant differences were observed among the three interventions in their effects on muscle mass. Based on SUCRA rankings, cholecalciferol had the highest probability of being the most effective treatment for increasing muscle mass (*p* = 77%), followed by protein supplementation (*p* = 69.4%) (Figure 12F and Supplementary Table 4). Given the limited number of included studies and heterogeneity in follow-up time points, these NMA findings should be interpreted with caution.

### 3.6 Network meta-analysis of serum metabolic parameters

**Figure 13:**
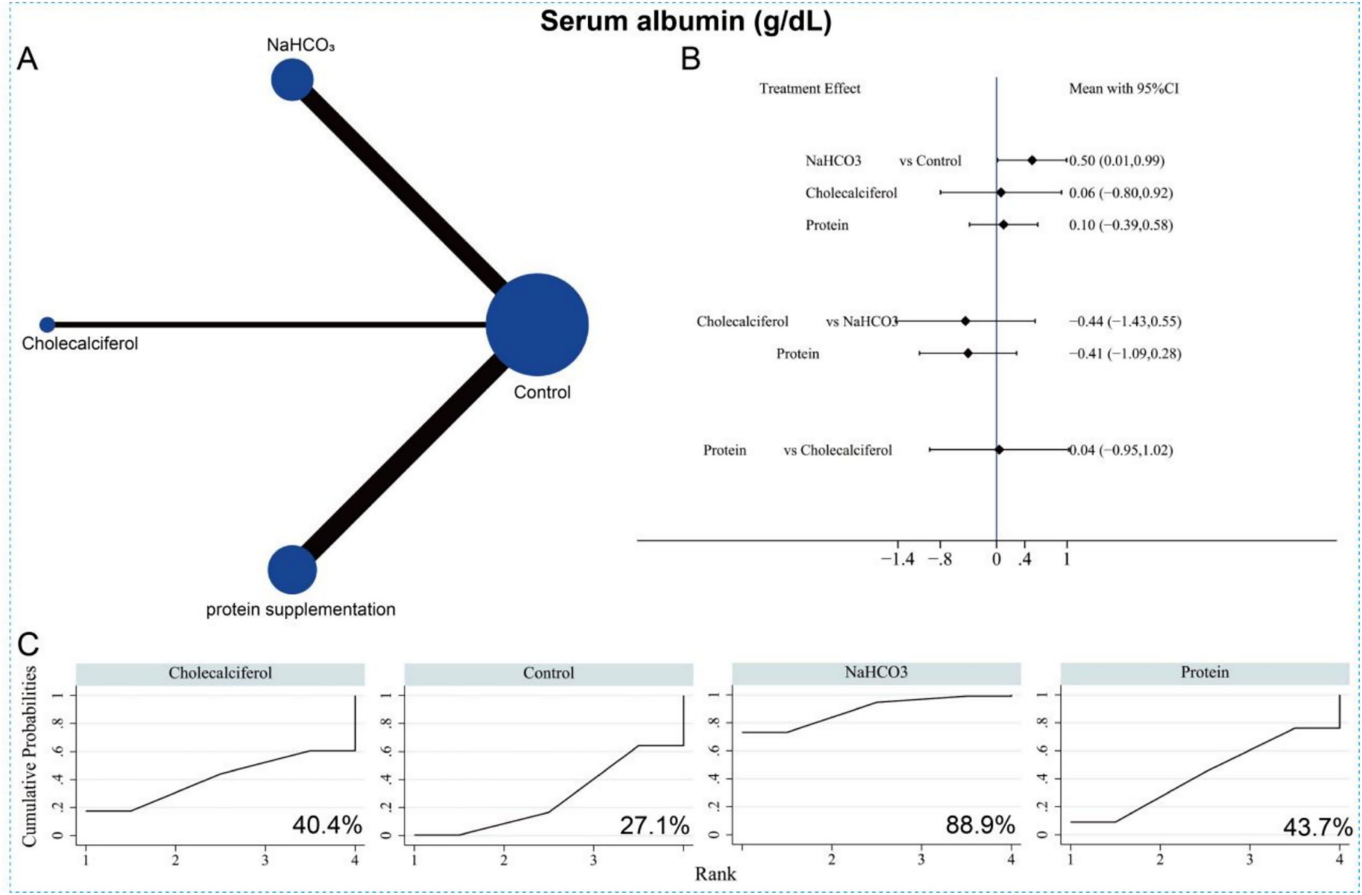
Network meta-analysis of the three different interventions on serum albumin. **(A)** Evidence network diagram of serum albumin. **(B)** Forest plot showing comparative evidence for three different interventions. **(C)** SUCRA curve and area under the curve (%) for serum albumin after three different interventions.

The NMA further compared the effects of different interventions on serum metabolic parameters to identify the most effective approach. Fourteen studies (15 datasets) involving 1,280 patients reported serum albumin levels in relation to the three interventions. The network evidence plot is shown in Figure 13A. Compared with the control group, sodium bicarbonate significantly increased serum albumin levels (SMD = 0.50, 95% CI = 0.01 to 0.99) (Figure 13B). However, no significant differences were observed among the three interventions in their effects on increasing serum albumin. Based on SUCRA rankings, sodium bicarbonate had the highest probability of being the most effective treatment for improving serum albumin (*p* = 88.9%), followed by protein supplementation (*p* = 43.7%) (Figure 13C and Supplementary Table 5).

**Figure 14:**
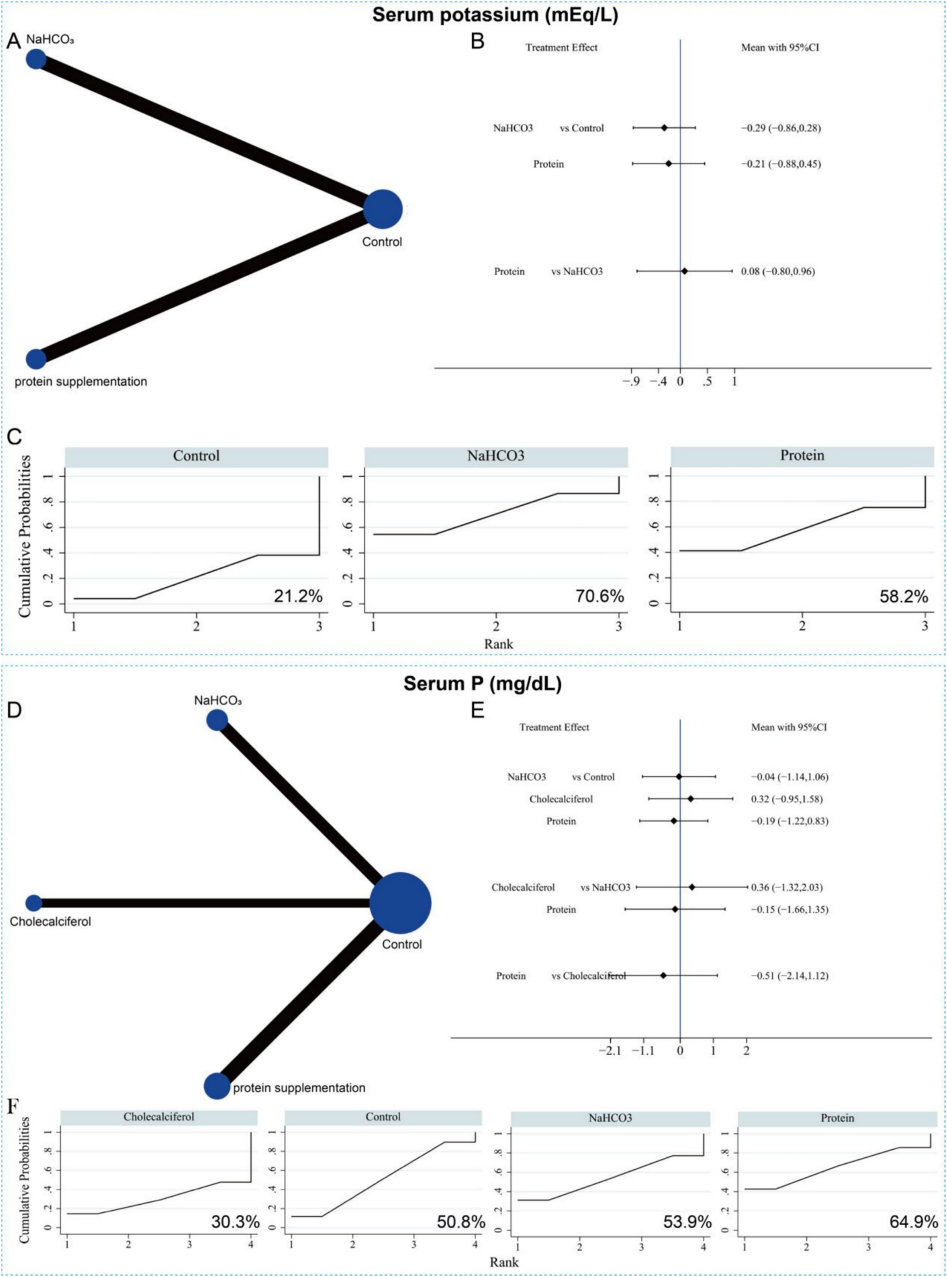
Network meta-analysis of the different interventions on serum potassium. **(A)** Evidence network diagram of serum potassium. **(B)** Forest plot showing comparative evidence for sodium bicarbonate and protein supplementation. **(C)** SUCRA curve and area under the curve (%) for serum potassium after different interventions. **(D)** Evidence network diagram of serum P. **(E)** Forest plot showing comparative evidence for three different interventions. **(F)** SUCRA curve and area under the curve (%) for serum P after three different interventions.

Eight studies involving 779 patients reported serum potassium levels following two interventions (sodium bicarbonate and protein supplementation). The network evidence plot is shown in Figure 14A. Compared with the control group, neither intervention significantly reduced serum potassium levels. No significant difference was observed between the two interventions either (Figure 14B). According to SUCRA rankings, sodium bicarbonate had the highest probability of being the most effective treatment for lowering serum potassium (*p* = 70.6%), followed by protein supplementation (*p* = 58.2%) (Figure 14C and Supplementary Table 6). Twelve studies, involving 751 patients, reported serum phosphorus levels following the three interventions. The network evidence plot is presented in Figure 14D. Compared with the control group, none of the interventions significantly reduced serum phosphorus levels. Likewise, no significant differences were found among the three interventions in their effects on serum phosphorus (Figure 14E). SUCRA rankings indicated that protein supplementation was most likely to be the most effective intervention for lowering serum phosphorus (*p* = 64.9%), followed by sodium bicarbonate (*p* = 53.9%) (Figure 14F and Supplementary Table 7).

**Figure 15:**
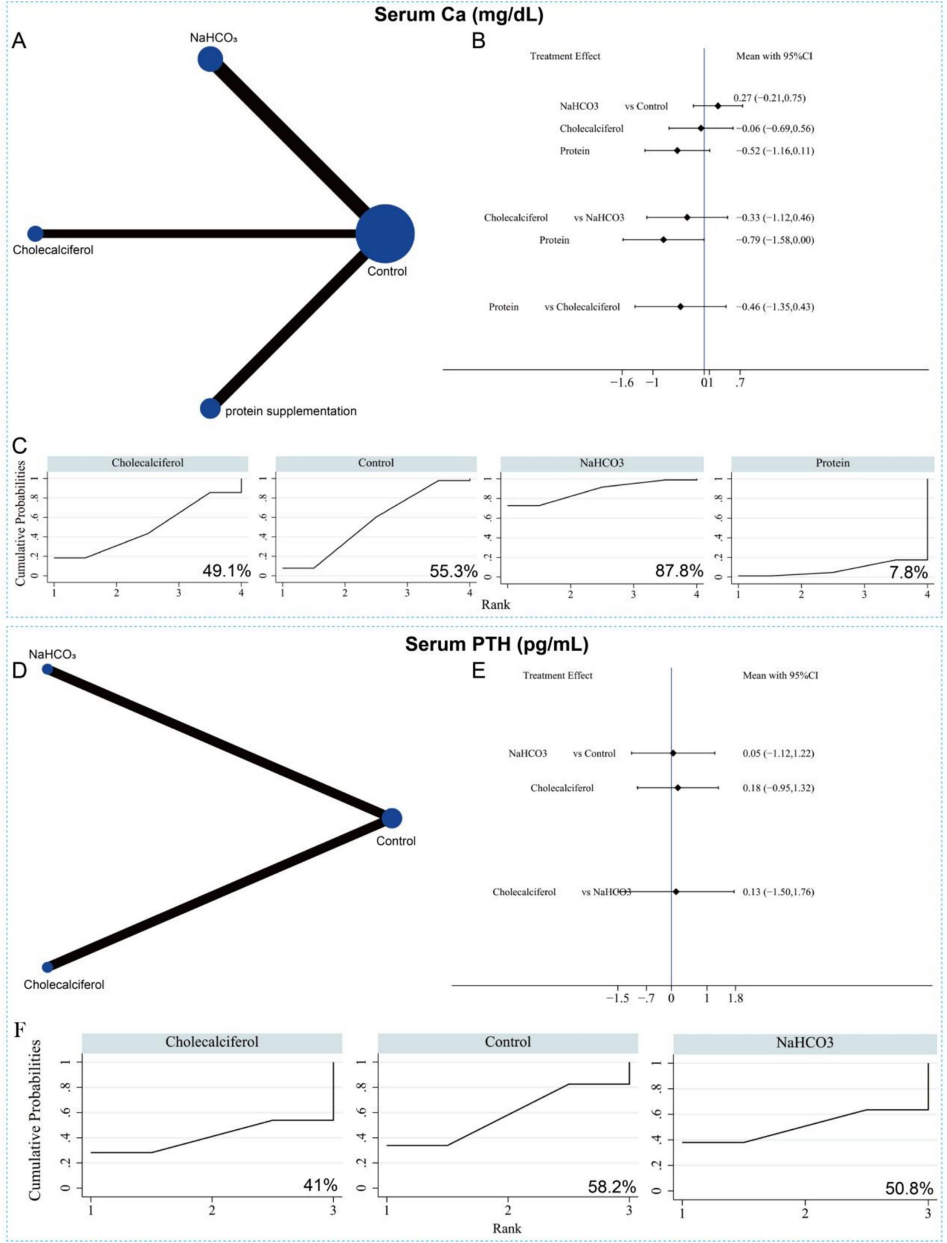
Network meta-analysis of the different interventions on serum *Ca.* **(A)** Evidence network diagram of serum *Ca.* **(B)** Forest plot showing comparative evidence for three different interventions *n*. **(C)** SUCRA curve and area under the curve (%) for serum Ca after different interventions. **(D)** Evidence network diagram of serum PTH. **(E)** Forest plot showing comparative evidence for sodium bicarbonate and cholecalciferol. **(F)** SUCRA curve and area under the curve (%) for serum PTH after two different interventions.

Additionally, 12 studies involving 977 patients reported serum calcium levels in relation to the three interventions. The network evidence plot is shown in Figure 15A. Compared with the control group, none of the interventions significantly increased serum calcium levels. Likewise, no significant differences were observed among the three interventions (Figure 15B). Based on SUCRA rankings, sodium bicarbonate had the highest probability of being the most effective treatment for increasing serum calcium (*p* = 87.8%) (Figure 15C and Supplementary Table 8). Only four studies reported serum PTH levels for two interventions (sodium bicarbonate and cholecalciferol) (Figure 15D). NMA results showed no significant difference in serum PTH levels between the two interventions (Figure 15E). According to SUCRA rankings, neither sodium bicarbonate nor cholecalciferol was likely to be the most effective intervention for reducing serum PTH, ranking second (*p* = 50.8%) and third (*p* = 41.0%), respectively (Figure 15F and Supplementary Table 9). Given the small sample sizes and limited number of studies, these NMA results should be interpreted with caution. In summary, cholecalciferol and sodium bicarbonate may play positive roles in increasing muscle mass and serum albumin levels, respectively, but show limited effects on other biochemical parameters.

### 3.7 Incidence of AEs

**Figure 16:**
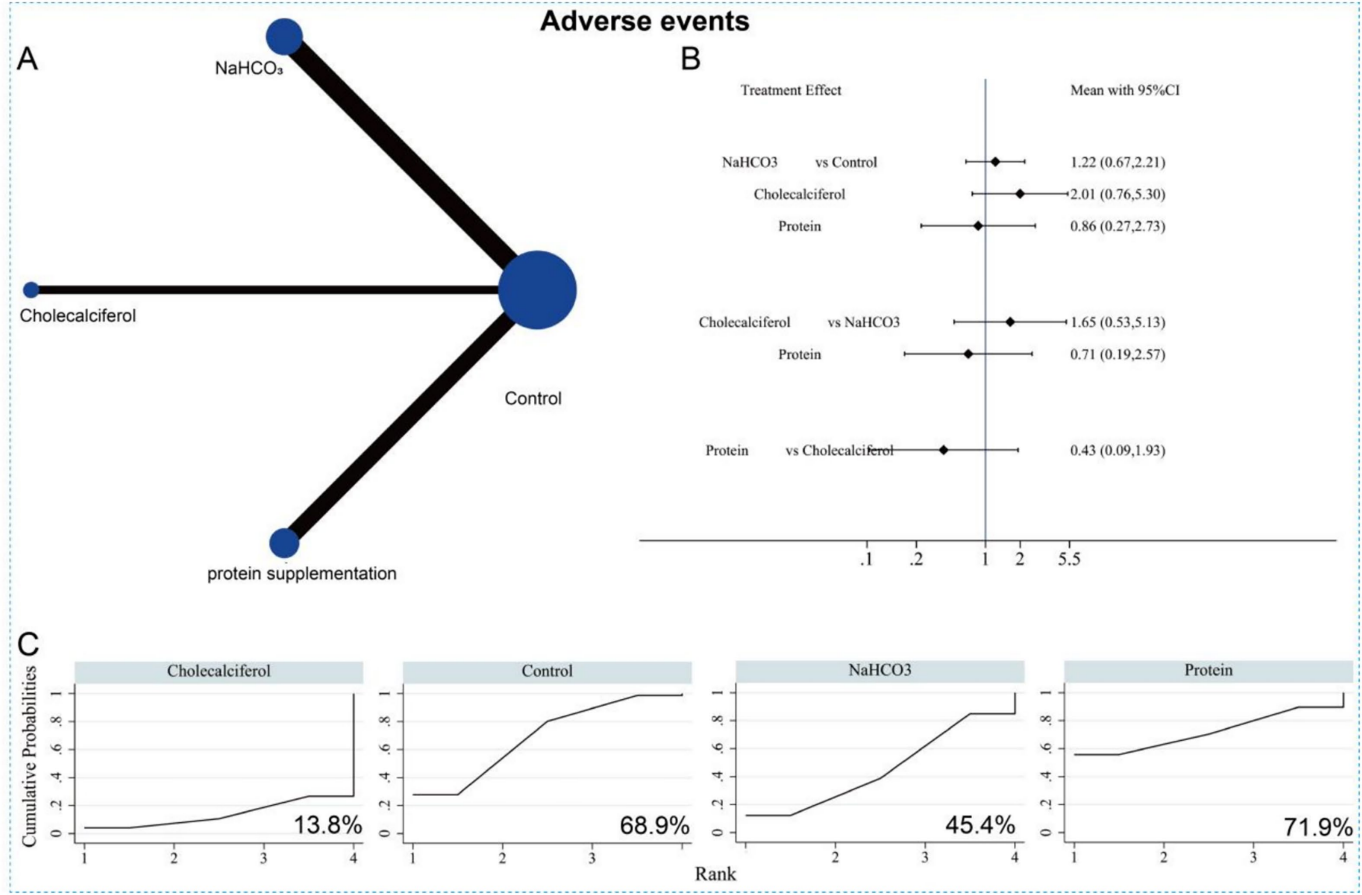
Network meta-analysis of the three different interventions on adverse events. **(A)** Evidence network diagram of adverse events. **(B)** Forest plot showing comparative evidence for three different interventions. **(C)** SUCRA curve and area under the curve (%) for adverse events after three different interventions.

For the safety assessment, 11 studies reported AE incidence across the three interventions, involving a total of 1,883 patients. The network evidence plot is shown in Figure 16A. The NMA indicated that, compared with the control group, none of the three interventions significantly reduced AE incidence. No significant differences were observed among the interventions either (Figure 16B). According to SUCRA rankings, protein supplementation was most likely to be the most effective option for reducing AE incidence (*p* = 71.9%) (Figure 16C and Supplementary Table 10). However, because of the limited number of studies and small sample sizes, as well as unclear AE definitions in the included literature, these NMA findings should be interpreted with caution.

### 3.8 Sensitivity analysis

In the conventional meta-analysis, sensitivity analyses were performed to evaluate the stability of pooled results for outcomes with at least five included studies. For six indicators, the sensitivity analyses showed that excluding any single study did not change the direction of the pooled effect estimates, indicating that the conventional meta-analysis results were robust (Supplementary Figure 16).

### 3.9 Publication bias

**Figure 17:**
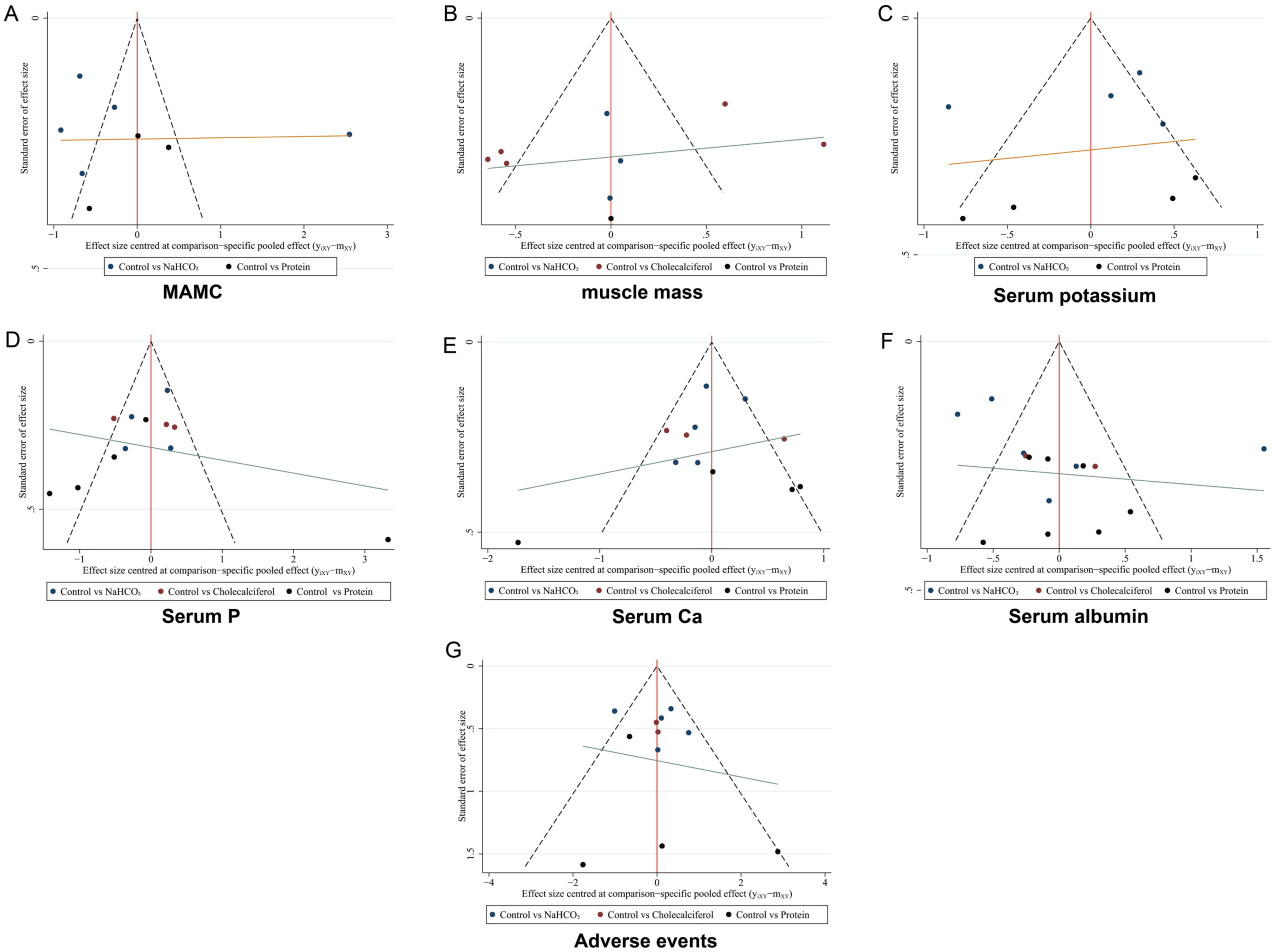
The funnel plot of network meta-analysis for each outcome indicator **(A)** MAMC; **(B)** Muscle mass; **(C)** Serum potassium; **(D)** Serum P; **(E)** Serum Ca; **(F)** Serum albumin; **(G)** Adverse events.

Furthermore, publication bias was assessed using funnel plots. Figures 17A–G present the funnel plots for different key outcomes, with no apparent asymmetry observed for any indicator. Overall, the results of the studies included appeared to be stable.

## 4 Discussion

During the progression of CKD, the ability to excrete non-volatile acids and reabsorb bicarbonate becomes impaired, often leading to acid retention and metabolic acidosis (13). This pathological state is associated with a range of severe consequences, including accelerated skeletal muscle catabolism, reduced bone mass, and increased mortality risk (59). Therefore, correcting metabolic acidosis, for instance through sodium bicarbonate administration, is considered a critical step in mitigating CKD complications (60). However, definitive evidence from well-designed, large-scale, multicenter clinical trials demonstrating that sodium bicarbonate therapy can significantly improve bone, muscle, and kidney health and enhance patient survival remains lacking (61, 62). On the other hand, emerging evidence suggests that cholecalciferol may also play a supportive role in muscle health among CKD patients (63) and has been identified as a predictor of mortality in dialysis patients (64). In addition, CKD patients, particularly those on dialysis, frequently suffer from protein–energy malnutrition, which is closely linked to poor prognosis (65). Protein supplementation may improve nutritional status and reduce mortality in hemodialysis patients (66). Nevertheless, due to the complex interplay of comorbidities, no meta-analysis has yet systematically compared the effects of these three interventions on muscle mass and metabolic disturbances in CKD patients.

In this NMA, clinical evidence on the use of sodium bicarbonate, cholecalciferol, and protein supplementation in patients with CKD was collected and synthesized, with the aim of providing the first comparative assessment of these interventions in improving muscle mass, serum metabolic disturbances, and the incidence of AEs. The analysis indicated that, compared with the control group, cholecalciferol may significantly increase muscle mass in CKD patients, whereas sodium bicarbonate primarily exerts a positive effect on raising serum albumin levels. Notably, the three interventions demonstrated distinct patterns of efficacy across muscle mass, serum metabolic parameters, and AE incidence. Cholecalciferol appears to be the most effective treatment for improving muscle mass, sodium bicarbonate may be optimal for reducing serum potassium and increasing serum albumin and calcium levels, while protein supplementation may be most effective for lowering serum phosphorus and AE incidence. Furthermore, direct evidence from conventional meta-analysis suggests that sodium bicarbonate intervention may also improve early serum HCO₃^−^ levels, as well as renal function and blood pressure at 24 months. Given these findings, along with the complex alterations in musculoskeletal health, nutrition, and acid–base balance in CKD patients, the potential of combination therapy to modulate the intricate microenvironment in CKD warrants consideration in future research. However, in light of the limited quantity of included evidence and relatively small sample sizes, further high-quality studies are required to validate these results.

As a commonly used alkaline agent for correcting metabolic acidosis in patients with CKD, the potential benefits of sodium bicarbonate primarily stem from its ability to restore acid–base balance, thereby attenuating muscle protein breakdown, improving bone metabolism, and modulating certain serum biochemical parameters. In the present meta-analysis, sodium bicarbonate significantly increased serum HCO₃^−^ levels in the short term, improved eGFR at 24 months, reduced early serum potassium levels, and showed potential for increasing serum albumin in the network meta-analysis. However, we found no statistically significant improvements in MAMC or sit-to-stand time compared with the control group, which is not entirely consistent with previous reports suggesting benefits for muscle anabolism. For example, one prior meta-analysis indicated that although correcting metabolic acidosis did not significantly affect serum albumin or blood urea nitrogen, it could improve muscle mass and physical function (26). Such discrepancies may relate to differences in baseline bicarbonate levels, intervention duration, or sample sizes among included populations.

Experimental studies have demonstrated that metabolic acidosis can increase osteoclast activity while reducing osteoblast activity, ultimately leading to bone loss (67). Several clinical trials have also shown that alkali therapy can significantly improve bone mineral density and calcium–phosphorus balance in postmenopausal women (68, 69). Nonetheless, our analysis did not detect significant improvements in bone metabolism–related markers such as serum calcium, phosphorus, or PTH with sodium bicarbonate treatment. This inconsistency may be partly attributable to the high sodium load, which could further promote protein catabolism and bone resorption (70). Importantly, recent research suggests that long-term sodium bicarbonate use, due to its sodium load, may contribute to fluid retention and elevated blood pressure (71). Consistent with this, we observed an increase in systolic blood pressure at 24 months following sodium bicarbonate intervention, underscoring the need to consider sodium-related risks. Overall, while sodium bicarbonate offers value in correcting acid–base imbalances and improving certain serum metabolic parameters, its clinical effects on muscle mass and bone metabolism remain uncertain. Individualized assessment and long-term safety monitoring are warranted, and future large-scale, long-duration randomized controlled trials are needed to clarify the balance between its multisystem benefits and potential risks.

Previous studies have shown that metabolic acidosis seems to accelerate the progression of CKD and is associated with muscle mass loss and more rapid deterioration of kidney function in CKD patients (72, 73). Early correction of metabolic acidosis may be beneficial in delaying CKD progression and preserving kidney function (74). However, in advanced CKD patients, the therapeutic effects of sodium bicarbonate may be influenced by various comorbidities, such as bone mineral metabolism disorders and cardiovascular diseases. For example, the BiCARB trial showed that correcting metabolic acidosis in patients with stage 4 or 5 CKD may be harmful and not cost-effective (75). Although there are fewer studies, our subgroup analysis results indicate that sodium bicarbonate supplementation can improve serum bicarbonate levels in patients with stage 3–4 and stage 4–5 CKD. Similarly, Di Iorio et al. (40) found that for stage 3–5 CKD patients, the use of sodium bicarbonate to treat metabolic acidosis is safe and can improve kidney function and patient survival. Therefore, future studies should consider conducting more detailed analyses in CKD patients at different stages to evaluate stage-specific intervention effects. As for the impact of sodium bicarbonate intervention dosage on CKD patient outcomes, there is no clear conclusion at present, with only a few studies describing dosage adjustments based on patients’ serum bicarbonate levels during the trial. For example, in the BiCARB trial, the starting dose of sodium bicarbonate was 1.5 g/day, and if serum bicarbonate levels were <22 mEq/L, the dose was increased to 3.0 g/day by month 3 (75). In the UBI study, the goal was to adjust serum bicarbonate levels to 24–28 mEq/L, meaning the intervention dose was personalized based on the patient’s serum bicarbonate levels (40). Although subgroup analyses show that the groups with a dose ≤ 0.3 mEq/kg, dose > 0.3 mEq/kg, and three times a day subgroups improved serum HCO₃^−^ levels, the limited number of studies and small sample sizes weaken the reliability of the subgroup analysis results. Therefore, future studies should further standardize the intervention protocols to enhance the comparability of results.

A noteworthy finding in this analysis is that the sodium bicarbonate group showed an increase in systolic blood pressure at 24 months, although no significant differences were observed at earlier time points. The increase in systolic blood pressure may be related to the sodium content of sodium bicarbonate treatment. Since sodium bicarbonate is a source of sodium, its use may lead to sodium retention, which in turn increases blood volume and raises blood pressure (76, 77). This is particularly concerning for patients with existing hypertension, as hypertension is a common comorbidity in CKD patients and in heart failure patients who are more sensitive to sodium load and fluid retention. Previous studies have shown that excessive sodium intake exacerbates hypertension and increases the risk of cardiovascular events, particularly in vulnerable populations such as CKD patients. For example, a study by Wesson et al. (57) demonstrated that the use of sodium-based alkalinizing therapy in CKD patients could lead to elevated blood pressure due to sodium retention. Additionally, the trial by Witham et al. (58) highlighted the potential risks of sodium load in CKD patients, particularly its effects on blood pressure and fluid balance. Given these concerns, it is crucial to consider the sodium content of sodium bicarbonate when prescribing this treatment, especially for CKD patients with comorbid hypertension or heart failure. Clinicians should carefully monitor blood pressure and fluid status during treatment, and further research is needed to assess the long-term cardiovascular effects of sodium bicarbonate, particularly in patients with pre-existing cardiovascular disease.

The findings of this study indicate that cholecalciferol demonstrated a certain advantage in improving muscle mass in the NMA. This effect may be attributable to the ability of active vitamin D to regulate mitochondrial function in skeletal muscle, promote oxidative phosphorylation, and activate the mTORC1 signaling pathway, thereby enhancing protein synthesis and inducing muscle hypertrophy (78, 79). Previous studies have also reported significant benefits of vitamin D supplementation for muscle strength (80) and increases in muscle fiber cross-sectional area in rat models (81). However, in the present study, direct evidence did not show significant improvements with cholecalciferol in MAMC, bone metabolism markers (serum calcium, phosphorus, PTH), or serum 25(OH)D levels. These discrepancies may be related to differences in intervention dose, form, duration, and baseline vitamin D status of participants. Future research should more precisely define the target population, optimize intervention protocols, and further explore the relationship between cholecalciferol use and muscle function improvement. Regarding protein supplementation, although theoretically it can improve negative nitrogen balance, facilitate muscle recovery, and compensate for protein loss during dialysis, our results showed no significant advantage in improving muscle mass, muscle function, or major biochemical markers. Previous clinical trials have also produced inconsistent findings, likely due to poor adherence, small sample sizes, short intervention durations, and the presence of multiple comorbidities (31, 82). Moreover, serum albumin, a commonly used marker of nutritional status, can be substantially influenced by fluid status and inflammatory responses (83, 84), which may partly explain why protein supplementation in our analysis did not significantly increase serum albumin levels and highlights its limitations in reflecting true nutritional improvement. Therefore, the role of protein supplementation in CKD still requires further high-quality studies to clarify its actual effects on nutritional optimization and muscle preservation.

Although this study primarily focused on the improvement of muscle mass, we also recognize that functional measures (such as grip strength, gait speed, and physical function) are important indicators for assessing the quality of life in CKD patients. Previous systematic reviews have shown that CKD patients with lower grip strength have a significantly higher risk of all-cause mortality (85, 86). Furthermore, improvements in gait speed and physical function are closely related to the quality of life. For example, a recent meta-analysis found that CKD patients with slower gait speed have a significantly increased risk of death (87). Physical function (such as the 6-min walk test and sit-to-stand test) is closely associated with quality of life, and exercise interventions can significantly improve physical function and quality of life in CKD patients (88, 89). In our analysis, only two studies reported the impact of sodium bicarbonate intervention on sit-to-stand time (5 repetition) in CKD patients, and no reliable conclusions could be drawn. Therefore, future research should further explore the changes in these functional indicators, especially the long-term impact of interventions on quality of life. A comprehensive consideration of these functional measures will help provide a more thorough evaluation of the clinical benefits of CKD interventions and offer a basis for developing more optimized treatment strategies.

## 5 Limitations

Although this meta-analysis is the first to compare the effects of three interventions on muscle mass, metabolic disturbances, and AE incidence in patients with CKD, and included stratified pooled analyses based on follow-up time points, several limitations remain. Firstly, the number of studies included in this research is limited, with many results based on only a few studies, and the sample sizes of these studies are generally small. This may affect the robustness of the results and the generalizability of the conclusions. Secondly, there was considerable heterogeneity among the included studies in terms of participant characteristics, intervention doses, and dosing frequencies, which could affect the accuracy of effect estimates. Thirdly, a substantial proportion of the included trials had open-label designs, and reporting on randomization, allocation concealment, and blinding was often incomplete, potentially introducing selection and measurement biases. Fourthly, validated minimal clinically important differences (MCIDs) are lacking for most of the surrogate outcomes examined in this network meta-analysis, particularly muscle mass indices and biochemical markers in CKD populations. This limitation restricts our ability to directly determine whether the observed effect sizes correspond to clinically meaningful benefits for patients. Although most studies reported the sex distribution of participants, the majority did not provide sex-stratified results for serum metabolic parameters, muscle-related outcomes, physical function, or quality-of-life measures. Therefore, this NMA could not reliably assess sex-based differences across interventions. Additionally, although serum creatinine and magnesium are important biochemical markers associated with CKD progression, most of the included studies reported renal function using eGFR rather than serum creatinine, and very few studies reported serum magnesium levels. Therefore, these parameters could not be included in the pooled analysis. Finally, some studies had small sample sizes and short intervention durations, making it difficult to adequately assess long-term outcomes and the incidence of adverse events. Therefore, we recommend interpreting the AE incidence results of this analysis with caution.

## 6 Conclusion

The findings of this NMA indicated that cholecalciferol may have a certain advantage in improving muscle mass, while sodium bicarbonate appeared more effective in increasing serum albumin, enhancing HCO₃^−^ and potassium levels in the early stage, and exerting some influence on renal function and blood pressure during long-term follow-up. Given the limited number of included studies, small sample sizes, and considerable heterogeneity in intervention protocols, these conclusions should be further validated in future large-scale, rigorously designed RCTs.
