# Supplementary material for: Effects of sodium bicarbonate, cholecalciferol, and protein supplementation interventions on muscle mass and metabolic disturbances in patients with chronic kidney disease: a systematic review and network meta-analysis
Source: Front Nutr. 2026 Apr 8;13:1698991. doi: 10.3389/fnut.2026.1698991 (PMC13099916; doi:10.3389/fnut.2026.1698991)
Supplement: Supplementary file 1 [file Supplementary_file_1.docx]

**Supplementary Materials**

| **Supplementary Table 1**. Search strategies in four main databases (Search Cut-off Date: July 1, 2025). | | | |
| --- | --- | --- | --- |
| **Databases** | **No.** | **Query** | **Results** |
| **Pubmed** | #1 | “Cholecalciferol” OR “Vitamin D3” OR “Cholecalciferols” OR “Calcifediol” OR “Sodium Bicarbonate” OR “Baking Soda” OR “Carbonic Acid Monosodium Salt” OR “Sodium Hydrogen Carbonate” OR “Protein” OR “Protein Supplement” OR “Amino Acid” OR “Oral Nutritional Supplement” | 19,909 |
|  | #2 | “Kidney Disease” OR “Chronic Kidney Disease” OR “CKD” OR “Renal Insufficiency” OR “Renal Dialysis” OR “Chronic Kidney Failure” OR “Kidney Failure” OR “ESRD” | 331,903 |
|  | #3 | “Metabolic Disturbance” OR “Serum Albumin” OR “Serum Potassium” OR “Serum Calcium” OR “Serum Phosphate” OR “Parathyroid Hormone” OR “Muscle” OR “Muscle Mass” OR “Muscle Function” OR “Mid-arm Muscle Circumference” OR “Bone Mineral Density” OR “Lean Body Mass” OR “Leg Lean Mass” | 1,228,135 |
|  | #4 | “Controlled clinical trial” OR “randomized controlled trial” OR “RCT” | 823,208 |
|  | #5 | #1 AND #2 AND #3 AND #4 | **635** |
| **Cochrane Library** | #6 | (Cholecalciferol):ab,ti,kw OR (Vitamin D3):ab,ti,kw OR (Cholecalciferols):ab,ti,kw OR (Calcifediol):ab,ti,kw OR (Sodium Bicarbonate):ab,ti,kw OR (Baking Soda):ab,ti,kw OR (Carbonic Acid Monosodium Salt):ab,ti,kw OR (Sodium Hydrogen Carbonate):ab,ti,kw OR (Protein):ab,ti,kw OR (Protein Supplement):ab,ti,kw OR (Amino Acid):ab,ti,kw OR (Oral Nutritional Supplement):ab,ti,kw | 119,538 |
|  | #7 | (Kidney Disease):ab,ti,kw OR (Chronic Kidney Disease):ab,ti,kw OR (CKD):ab,ti,kw OR (Renal Insufficiency):ab,ti,kw OR (Renal Dialysis):ab,ti,kw OR (Chronic Kidney Failure):ab,ti,kw OR (Kidney Failure):ab,ti,kw OR (ESRD):ab,ti,kw | 54,494 |
|  | #8 | (Metabolic Disturbance):ab,ti,kw OR (Serum Albumin):ab,ti,kw OR (Serum Potassium):ab,ti,kw OR (Serum Calcium):ab,ti,kw OR (Serum Phosphate):ab,ti,kw OR (Parathyroid Hormone):ab,ti,kw OR (Muscle):ab,ti,kw OR (Muscle Mass):ab,ti,kw OR (Muscle Function):ab,ti,kw OR (Mid-arm Muscle Circumference):ab,ti,kw | 130,531 |
|  | #9 | (Controlled clinical trial):ab,ti,kw OR (randomized controlled trial):ab,ti,kw OR (RCT):ab,ti,kw | 869,586 |
|  | #10 | #6 AND #7 AND #8 AND #9 | **1336** |
| **Web of Science** | #11 | TS=(Cholecalciferol OR Vitamin D3 OR Cholecalciferols OR Calcifediol OR Sodium Bicarbonate OR Baking Soda OR Carbonic Acid Monosodium Salt OR Sodium Hydrogen Carbonate OR Protein OR Protein Supplement OR Amino Acid OR Oral Nutritional Supplement) | 4,569,489 |
|  | #12 | TS=(Kidney Disease OR Chronic Kidney Disease OR CKD OR Renal Insufficiency OR Renal Dialysis OR Chronic Kidney Failure OR Kidney Failure OR ESRD) | 341,966 |
|  | #13 | TS=(Metabolic Disturbance OR Serum Albumin OR Serum Potassium OR Serum Calcium OR Serum Phosphate OR Parathyroid Hormone OR Muscle OR Sodium Hydrogen Carbonate OR Muscle Mass OR Muscle Function OR Mid-arm Muscle Circumference) | 1,063,853 |
|  | #14 | TS=(Controlled clinical trial OR randomized controlled trial OR RCT) | 676,043 |
|  | #15 | #11 AND #12 AND #13 AND #14 | **639** |
| **Embase** | #16 | ('Cholecalciferol':ab,ti,kw OR 'Vitamin D3':ab,ti,kw OR 'Cholecalciferols':ab,ti,kw OR 'Calcifediol':ab,ti,kw OR 'Sodium Bicarbonate':ab,ti,kw OR 'Baking Soda':ab,ti,kw OR 'Carbonic Acid Monosodium Salt':ab,ti,kw OR 'Sodium Hydrogen Carbonate':ab,ti,kw OR 'Protein':ab,ti,kw OR 'Protein Supplement':ab,ti,kw OR 'Amino Acid':ab,ti,kw OR 'Oral Nutritional Supplement':ab,ti,kw) | 4,191,329 |
|  | #17 | ('Kidney Disease':ab,ti,kw OR 'Chronic Kidney Disease':ab,ti,kw OR 'CKD':ab,ti,kw OR 'Renal Insufficiency':ab,ti,kw OR 'Renal Dialysis':ab,ti,kw OR 'Chronic Kidney Failure':ab,ti,kw OR 'Kidney Failure':ab,ti,kw OR 'ESRD':ab,ti,kw) | 318,606 |
|  | #18 | ('Metabolic Disturbance':ab,ti,kw OR 'Serum Albumin':ab,ti,kw OR 'Serum Potassium':ab,ti,kw OR 'Serum Calcium':ab,ti,kw OR 'Serum Phosphate':ab,ti,kw OR 'Parathyroid Hormone':ab,ti,kw OR 'Muscle':ab,ti,kw OR 'Sodium Hydrogen Carbonate':ab,ti,kw OR 'Muscle Mass':ab,ti,kw OR 'Muscle Function':ab,ti,kw OR 'Mid-arm Muscle Circumference':ab,ti,kw) | 1,252,538 |
|  | #19 | ('Controlled clinical trial':ab,ti,kw OR 'randomized controlled trial':ab,ti,kw OR 'RCT':ab,ti,kw) | 303,859 |
|  | #20 | #16 AND #17 AND #18 AND #19 | **162** |
| **Total** | #21 | #5 AND #10 AND #15 AND #20 | **2772** |

| **Supplementary Table 2**. Main characteristics of all studies for sodium bicarbonate vs control. | | | | | | | | | | | | | | | | | | | | | | | | | | | | | | | |
| --- | --- | --- | --- | --- | --- | --- | --- | --- | --- | --- | --- | --- | --- | --- | --- | --- | --- | --- | --- | --- | --- | --- | --- | --- | --- | --- | --- | --- | --- | --- | --- |
| **Author** | **Year** | **Country** | **Study design** | **Study vs Control** | **Age (Study vs Control, Years ± SD, range)** | **Number of female/male (Study vs Control)** | **BMI (kg/m2)(Study vs Control)** |  | **blood pressure(mmHg)** | |  | **Diagnosis etiology of CKD** | | | | | |  | **Medication use** | | | |  | **Laboratory characteristics (baseline)** | | |  | **sodium bicarbonate** | | | |
|  |  |  |  |  |  |  |  |  | **Systolic blood pressure** | **Diastolic blood pressure** |  | **Hypertension** | **Kidney disease** | **Heart failure** | **Peripheral and coronary artery disease** | **Diabetes** | **Other causes** |  | **Diuretic** | **ACE inhibitor or ARB** | **β-Blocker** | **other** |  | **eGFR （ml/min/1.73 m^2^)** | **Serum bicarbonate (mEq/L)** | **Serum potassium (mEq/L)** |  | **Route** | **dose** | **Time** | **HCO3 target value（mEq/L）** |
| Alva et al [37] | 2020 | India | RCT | sodium bicarbonate vs standard therapy | NA | 10/23 vs 9/25 | 22.40 vs 23.48 |  | NA | NA |  | 7 vs 8 | NA | 3 vs 3 | 4 vs 6 | 6 vs 6 | 13 vs 11 |  | NA | NA | NA | NA |  | NA | 16.62±3.05 vs 16.84 ±2.17 | NA |  | oral | 0.37 mEq/kg | three times a day | >23 |
| De Brito-Ashurst et al [39] | 2009 | UK | RCT | sodium bicarbonate vs standard therapy | 54.78 ± 2.56 vs 54.77±2.34 | 15/52 vs 15/51 | NA |  | 124.0 ± 1.3 vs 123.7 ± 1.2 | 76.1 ± 1.5 vs 75.4 ± 1.9 |  | 29 vs 26 | 11 vs 17 | NA | NA | 37 vs 36 | 10 vs 14 |  | 70 vs 67 | 50 vs 48 | 19 vs 17 | 59 vs 57 |  | 20.12 ± 6.47 vs 20.70 ± 5.55 | 19.8 ± 2.2 vs 19.9 ± 1.5 | NA |  | oral | 0.28 mEq/kg | three times a day | >23 |
| Di Iorio et al [40] | 2019 | Italy | RCT | sodium bicarbonate vs standard care | 67.6±15.1 vs 68.1±14.7 | 142/234 vs 140/224 | 27.7±4.6 vs 28.2±4.6 |  | 129±19 vs 128±18 | 74±11 vs 73±8 |  | 62 vs 69 | 87 vs 85 | 14 vs 22 | 171 vs 174 | 128 vs 99 | 85 vs 89 |  | 348 vs 312 |  |  | 128 vs 98 |  | 33.4±12.4 vs 36.9±10.8 | 21.7±2.6 vs 21.4±2.1 | 4.9±0.5 vs 4.9±0.6 |  | oral | 0.6 mEq/kg | NA | 24-28 |
| Dubey et al [41] | 2020 | India | RCT | sodium bicarbonate vs standard therapy | 50.12 ± 11.6 vs 50.30±11.4 | 26/68 vs 28/66 | 21.2±2.96 vs 21.3±3.46 |  | 130±17.31 vs 131±19.78 | 82±12.36 vs 83±12.36 |  | NA | 34 vs 27 | NA | NA | 14 vs 15 | 46 vs 52 |  | 36 vs 43 | 29 vs 23 | 20 vs 29 | 2 vs 11 |  | 29.2±10.63 vs 31.5±11.12 | 18.1±2.22 vs 18.1±2.47 | NA |  | oral | 0.5 mEq/kg | NA | 24-28 |
| Jeong et al [44] | 2014 | Korea | Cohort study | sodium bicarbonate vs standard care | 53.3±13.5 vs 55.8±12.7 | 14/26 vs 19/21 | 23.10±3.26 vs 23.03±2.87 |  | 139.1±16.2 vs 141.1±20.9 | 76.07±11.1 vs 81.76±10.3 |  | 16 vs 15 | 9 vs 12 | NA | NA | 11 vs 10 | 4 vs 3 |  | NA | NA | NA | NA |  | 16.7±6.1 vs 17.7±6.4 | NA | 5.25±0.71 vs 5.44±0.63 | | oral | 0.58 mEq/kg | three times a day | >22 |
| Kendrick et al [47] | 2018 | Colorado | RCT | sodium bicarbonate vs standard therapy | NA | 10/10 vs 10/10 | NA |  | NA | NA |  | NA | NA | NA | NA | NA | NA |  | NA | NA | NA | NA |  | 26±8.0 | 19.3±2.9 vs 19.7±2.3 | NA |  | oral | 0.4 mEq/kg | three times a day | >23 |
| Kittiskulnam et al [48] | 2020 | Thailand | RCT | sodium bicarbonate vs placebo | 59.0±12.5 vs 63.3±5.4 | 9/12 vs 9/12 | NA |  | 147.0±22.4 vs 156.6±21.9 | 78.2±10.5 vs 79.2±11.2 |  | 5 vs 1 | 1 vs 6 | NA | NA | 12 vs 13 | 3 vs 1 |  | 3 vs 5 | 14 vs 13 | NA | 3 vs 7 |  | 36.4±15.3 vs 28.4±11.9 | 21.2±2.0 vs 20.9±2.3 | 4.4±0.5 VS 4.5±0.5 |  | oral | 0.3 mEq/kg | NA | 24-26 |
| Mathur et al [49] | 2006 | India | RCT | sodium bicarbonate vs placebo | 37.5 ± 17 vs 43.5 ± 10.5 | 8/12 vs 7/13 | NA |  | 136 ± 7 vs 132 ± 6 | 86 ± 5 vs 88 ± 4 |  | NA | NA | NA | NA | NA | NA |  | NA | NA | NA | NA |  | NA | 19.49 ± 5.51 vs 19.35 ± 3.74 | NA |  | oral | 1.2 mEq/kg | three times a day | 22-26 |
| Melamed et al [50] | 2020 | USA | RCT | sodium bicarbonate vs placebo | 60.3±14.1 vs 61.6±10.9 | 38/36 vs 42/33 | 33.4±7.7 vs 33.8±8.4 |  | 138±18 vs 136±16 | 75±9 vs 76±11 |  | 68 vs 71 | NA | 8 vs 8 | NA | 49 vs 43 | NA |  | 42 vs 50 | 58 vs 58 | NA | 40 vs 43 |  | 36.4±11.4 vs 36.2±11.1 | 24.0±2.2 vs 24.1±2.6 | 4.5±0.5 vs 4.6±0.5 |  | oral | 0.4 mEq/kg | NA | NA |
| Wesson et al [57] | 2019 | USA | RCT | sodium bicarbonate vs placebo | 62.9±12.6 vs 63.2±12.1 | 50/74 vs 33/60 | 28.8±4.2 vs 28.3±4.1 |  | 136.1±9.1 vs 136.5±9.1 | NA |  | 37 vs 29 | 20 vs 15 | NA | NA | 17 vs 19 | 9 vs 2 |  | 74 vs 59 | 83 vs 76 | 57 vs 49 | 66 vs 52 |  | 29.2±6.3 vs 27.8±5.4) | 17.3±1.4 vs 17.3±1.5 | 4.9±0.6 vs 4.9±0.6 |  | oral | 6 g/day | twice a day | 22–29 |
| Witham et al [58] | 2020 | UK | RCT | sodium bicarbonate vs placebo | 73.9±7.6 vs 74.0±6.6 | 42/110 vs 44/104 | 28.9±4.5 vs 28.3±4.6 |  | 143±18 vs 143±18 | 75±11 vs 73±10 |  | 37 vs 40 | 20 vs 20 | NA | 19 vs 21 | 23 vs 23 | 83 vs 85 |  | NA | 105 vs 91 | NA | 32 vs 28 |  | 19.7±6.5 vs 18.2±6.4 | 20.6±2.6 vs 20.1±2.5 | 4.9±0.5 vs 4.9±0.5 |  | oral | 0.22 mEq/kg | three times a day | >22 |
| NA: not application; RCTs: randomized controlled trials. | | | | | | | | | | | | | | | | | | | | | | | | | | | | | | | |

| **Supplementary Table 3**. Main characteristics of all studies for cholecalciferol vs control. | | | | | | | | | | | | | | | | | | | | | | | | | | | |
| --- | --- | --- | --- | --- | --- | --- | --- | --- | --- | --- | --- | --- | --- | --- | --- | --- | --- | --- | --- | --- | --- | --- | --- | --- | --- | --- | --- |
| **Author** | **Year** | **Country** | **Study design** | **Study vs Control** | **Age (Study vs Control, Years ± SD, range)** | **Number of female/male (Study vs Control)** | **BMI (kg/m^2^) (Study vs Control)** |  | **blood pressure(mmHg)** | |  | **Diagnosis etiology of CKD** | | | |  | **Medication use** | |  | **Laboratory characteristics (baseline)** | | | |  | **cholecalciferole** | | |
|  |  |  |  |  |  |  |  |  | **Systolic blood pressure** | **Diastolic blood pressure** |  | **Kidney disease** | **Peripheral and coronary artery disease** | **Diabetes** | **Other causes** |  | **ACE inhibitor or ARB** | **other** |  | **plasma calcium（mmol/L）** | **phosphate（mmol/L）** | **PTH（pmol/L）** | **25(OH)D (nmol/L)** |  | **Route** | **dose** | **Time** |
| Singer et al [53] | 2018 | Australia | RCT | Cholecalciferol vs Placebo | 59.5 ± 15.6 vs 63.8 ±14.2 | 9/23 vs 13/23 | NA |  | 142.1 ± 18.8 vs 142.2 ± 19.4 | 74.9 ± 13.3 vs 71.6 ± 11.2 |  | NA | NA | NA | NA |  | NA | NA |  | 2.26±0.22 vs 2.33±0.12 | 1.65±0.71 vs 1.73±0.56 | 38.38±36.51 vs 34.88±33.75 | 33.9 ± 9.0 vs 33.8 ± 11.2 |  | oral | 50000 U | weekly |
| Wang et al [56] | 2024 | China | prospective cohort study | Cholecalciferol vs Placebo | 53.90±2.72 vs 58±3.19 | 61/61 vs 40/61 | 22.04±0.42 vs 21.55±0.23 |  | NA | NA |  | NA | NA | NA | NA |  | NA | NA |  | 2.13±0.04 vs 2.23±0.02 | 1.55±0.11 vs 1.53±0.11 | 329.36±33.48 vs 234.65±24.77 | 12.84±0.7 vs 13.37±0.81 |  | oral | 0.25 µg per day | once a day |
| Hewitt et al [43] | 2013 | Australia | RCT | Cholecalciferol vs Placebo | 60.31±3.89 vs 66.37±3.89 | 7/53 vs 17/43 | 26.6±6.4 vs 31.3±9.5 |  | NA | NA |  | NA | 30 vs 35 | 15 vs 18 | NA |  | NA | NA |  | 9.4±0.5 vs 9.4±0.6 | 5.4±1.6 vs 4.8±1.6 | 335±327 vs 222±224 | 18±5 vs 16±5 |  | oral | 50000 U | weekly |
| Jiampochaman et al [46] | 2025 | Thailand | RCT | Cholecalciferol vs Placebo | 57.6±11.8 vs 56.9±10.9 | 18/20 vs 16/22 | 24.8±4.3 vs 23.7±4.2 |  | 143.1±18.6 vs 138.2±25.2 | 80.8±11.6 vs 76.9±13.6 |  | 3 vs 3 | NA | 7 vs 12 | 18 vs 12 |  | 13 vs 9 | 6 vs 8 |  | 8.9±1.0 vs 8.8±1.0 | 5.1±1.4 vs 4.4±1.7 | 341.51±82.42 vs 372.50±146.02 | 15.1±6.4 vs 14.5±7.3 |  | oral | 20000 U | weekly |
| Mori et al [51] | 2013 | Japan | prospective cohort study | Cholecalciferol vs Placebo | 59.6±12.9 vs 57.7±14.6 | 24/18 vs 16/10 | 22.0±3.4 vs 22.1±3.4 |  | NA | NA |  | 36 vs 13 | NA | 2 vs 7 | 4 vs 6 |  | NA | NA |  | 9.5±1.0 vs 9.0±0.7 | NA | 273.2±174.5 vs 87.3±62.9 | NA |  | oral | NA | NA |
| NA: not application; RCTs: randomized controlled trials. | | | | | | | | | | | | | | | | | | | | | | | | | | | |

| **Supplementary Table 4**. Main characteristics of all studies for protein supplementation vs control. | | | | | | | | | | | | | | | | | | | | | |
| --- | --- | --- | --- | --- | --- | --- | --- | --- | --- | --- | --- | --- | --- | --- | --- | --- | --- | --- | --- | --- | --- |
| Author | Year | Country | Study design | Study vs Control | Age (Study vs Control, Years ± SD, range) | Number of female/male (Study vs Control) | BMI (kg/m2)(Study vs Control) |  | blood pressure(mmHg) | |  | Diagnosis etiology of ESRD | | | | |  | protein supplementation | | | |
|  |  |  |  |  |  |  |  |  | Systolic blood pressure | Diastolic blood pressure |  | Hypertension | Kidney disease | Peripheral and coronary artery disease | Diabetes | Other causes |  | Route | dose | Time | Type of protein |
| Jeong et al [45] | 2019 | USA | RCT | protein supplementation vs Control | 56.6 ± 13.0 vs 54.4 ± 12.3 | 15/23 vs 6/28 | 30.6±7.1 vs 31.5±7.6 |  | 144.6±23.1 vs 140.6±26.6 | 78.2±13.4 vs 81.4±15.2 |  | 24 vs 20 | 1 vs 6 | NA | 12 vs 13 | 8 vs 5 |  | oral | 30 g | Three times a week | whey protein |
| Calegari et al [38] | 2011 | Brazil | RCT | protein vs Control | 56.4±15.58 | 3/15 vs 3/15 | 22.28±2.32 vs 20.85±2.14 |  | NA | NA |  | NA | NA | NA | NA | NA |  | oral | NA | Three times a week | nutritional |
| González-Espinoza et al [42] | 2005 | Mexico | RCT | protein vs Control | 45.7±14.4 vs 47.6±17.4 | 5/8 vs 4/11 | NA |  | 163±30 vs 150±25 | 91±15 vs 85±18 |  | NA | NA | NA | 4 vs 6 | 9 vs 9 |  | oral | 15 g | Twice a day | egg-based albumin |
| Sahathevan et al [52] | 2018 | Malaysia | RCT | protein vs Control | 50.84±15.20 vs 42.14±14.57 | 20/17 vs 22/15 | 21.65±2.81 vs 21.22±2.35 |  | NA | NA |  | 30 vs 28 | NA | NA | 11 vs 8 | 4 vs 4 |  | oral | 15 g | Twice a day | whey protein |
| Teixidó-Planas et al [54] | 2005 | Spain | RCT | protein vs Control | 52.42±11.64 vs 53.69±12.00 | 13/17 vs 15/20 | NA |  | NA | NA |  | NA | 15 vs 12 | 5 vs 6 | 8 vs 7 | 7 vs 5 |  | oral | 200 mL | Once a day | protein drink |
| Tomayko et al [55] | 2015 | USA | RCT | protein vs Control | 57.0±4.8 vs 53.3±2.4 | 4/7 vs 5/10 | 31.3±2.2 vs 32.8±2.0 |  | NA | NA |  | NA | NA | NA | NA | NA |  | oral | 27 g | Three times a week | whey protein |
|  |  |  |  |  | 52.5±4.3 vs 53.3±2.4 | 5/7 vs 5/10 | 33.3±2.4 vs 32.8±2.0 |  | NA | NA |  | NA | NA | NA | NA | NA |  | oral | 27 g | Three times a week | soy protein |
| NA: not application; RCTs: randomized controlled trials. | | | | | | | | | | | | | | | | | | | | | |

| **Supplementary Table 5**. Newcastle-Ottawa Scale for risk of bias assessment of cohort studies included in the meta-analysis. | | | | | | | | | |
| --- | --- | --- | --- | --- | --- | --- | --- | --- | --- |
| Study | Selection | | | | Comparability | Exposure | | | Scores |
|  | Representativeness of Exposed Cohort | Selection of Nonexposed | Ascertainment of Exposure | Outcome Not Present at Start | Comparability between groups | Assessment of Outcome | Adequate Follow- Up Length | Adequacy of Follow-Up |  |
| Jeong 2014 | ★ | ▲ | ★ | ★ | ★★ | ★ | ★ | ▲ | 8 |
| Wang 2024 | ★ | ▲ | ★ | ★ | ★★ | ★ | ★ | ★ | 8.5 |
| Mori 2013 | ▲ | ★ | ▲ | ★ | ★★ | ★ | ★ | ★ | 8 |
| ▲: score of 0.5; ★: score of 1; ★★: score of 2 | | | | | | | | | |

| **Supplementary Table 6**. League table demonstrating the results of the network meta-analysis comparing the serum MAMC (cm) (SMD, 95% CI). | | | |
| --- | --- | --- | --- |
| **Treatment** | **NaHCO₃** | **Protein** | **Control** |
| **NaHCO₃** | 1 | -0.58 (-2.33,1.16) | -0.92 (-1.98,0.14) |
| **Protein** | 0.58 (-1.16,2.33) | 1 | -0.33 (-1.72,1.05) |
| **Control** | 0.92 (-0.14,1.98) | 0.33 (-1.05,1.72) | 1 |
| CI: confidence interval; MAMC: midarm muscle circumference; SMD: standardized mean difference. | | | |

| **Supplementary Table 7**. League table demonstrating the results of the network meta-analysis comparing the muscle mass (kg) (SMD, 95% CI). | | | | | |
| --- | --- | --- | --- | --- | --- |
| **Treatment** | **Cholecalciferol** | **Protein** | **NaHCO₃** | **Control** |  |
| **Cholecalciferol** | 1 | 0.02 (-1.51,1.55) | -0.47 (-1.43,0.50) | **-0.68 (-1.27,-0.09)** |  |
| **Protein** | -0.02 (-1.55,1.51) | 1 | -0.49 (-2.09,1.12) | -0.70 (-2.11,0.71) |  |
| **NaHCO₃** | 0.47 (-0.50,1.43) | 0.49 (-1.12,2.09)) | 1 | -0.21 (-0.98,0.55) |  |
| **Control** | **0.68 (0.09,1.27)** | 0.70 (-0.71,2.11) | 0.21 (-0.55,0.98) | 1 |  |
| CI: confidence interval; SMD: standardized mean difference. | | | | | |

| **Supplementary Table 8**. League table demonstrating the results of the network meta-analysis comparing the serum albumin (g/dL) (SMD, 95% CI). | | | | | |
| --- | --- | --- | --- | --- | --- |
| **Treatment** | **NaHCO₃** | **Protein** | **Cholecalciferol** | **Control** |  |
| **NaHCO₃** | 1 | -0.41 (-1.09,0.28) | -0.44 (-1.43,0.55) | **-0.50 (-0.99,-0.01)** |  |
| **Protein** | 0.41 (-0.28,1.09) | 1 | -0.04 (-1.02,0.95) | -0.10 (-0.58,0.39) |  |
| **Cholecalciferol** | 0.44 (-0.55,1.43) | 0.04 (-0.95,1.02) | 1 | -0.06 (-0.92,0.80) |  |
| **Control** | **0.50 (0.01,0.99)** | 0.10 (-0.39,0.58) | 0.06 (-0.80,0.92) | 1 |  |
| CI: confidence interval; SMD: standardized mean difference. | | | | | |

| **Supplementary Table 9**. League table demonstrating the results of the network meta-analysis comparing the serum potassium (mEq/L) (SMD, 95% CI). | | | | | |
| --- | --- | --- | --- | --- | --- |
| **Treatment** | **NaHCO₃** | **Protein** | **Control** |  |  |
| **NaHCO₃** | 1 | 0.08 (-0.80,0.96) | **0.29 (-0.28,0.86)** |  |  |
| **Protein** | -0.08 (-0.96,0.80) | 1 | 0.21 (-0.45,0.88) |  |  |
| **Control** | **-0.29 (-0.86,0.28)** | -0.21 (-0.88,0.45) | 1.00 |  |  |
| CI: confidence interval; SMD: standardized mean difference. | | |  |  |  |

| **Supplementary Table 10**. League table demonstrating the results of the network meta-analysis comparing the serum P (mg/dL) (SMD, 95% CI). | | | | | |
| --- | --- | --- | --- | --- | --- |
| **Treatment** | **Protein** | **Control** | **NaHCO₃** | **Cholecalciferol** |  |
| **Protein** | 1 | 0.15 (-1.35,1.66) | 0.19 (-0.83,1.22) | 0.51 (-1.12,2.14) |  |
| **Control** | -0.15 (-1.66,1.35) | 1 | 0.04 (-1.06,1.14) | 0.36 (-1.32,2.03) |  |
| **NaHCO₃** | -0.19 (-1.22,0.83) | -0.04 (-1.14,1.06) | 1 | 0.32 (-0.95,1.58) |  |
| **Cholecalciferol** | -0.51 (-2.14,1.12) | -0.36 (-2.03,1.32) | -0.32 (-1.58,0.95) | 1 |  |
| CI: confidence interval; SMD: standardized mean difference. | | | | |  |

| **Supplementary Table 11**. League table demonstrating the results of the network meta-analysis comparing the serum Ca (mg/dL) (SMD, 95% CI). | | | | | |
| --- | --- | --- | --- | --- | --- |
| **Treatment** | **NaHCO₃** | **Control** | **Cholecalciferol** | **Protein** |  |
| **NaHCO₃** | 1 | **-0.27 (-0.75,0.21)** | **-0.33 (-1.12,0.46)** | **-0.79 (-1.58,-0.00)** |  |
| **Control** | **0.27 (-0.21,0.75)** | 1 | -0.06 (-0.69,0.56) | **-0.52 (-1.16,0.11)** |  |
| **Cholecalciferol** | **0.33 (-0.46,1.12)** | 0.06 (-0.56,0.69) | 1 | -0.46 (-1.35,0.43) |  |
| **Protein** | **0.79 (0.00,1.58)** | **0.52 (-0.11,1.16)** | 0.46 (-0.43,1.35) | 1 |  |
| CI: confidence interval; SMD: standardized mean difference. | | | | |  |

| **Supplementary Table 12**. League table demonstrating the results of the network meta-analysis comparing the serum PTH (pg/mL) (SMD, 95% CI). | | | |
| --- | --- | --- | --- |
| **Treatment** | **Control** | **NaHCO₃** | **Cholecalciferol** |
| **Control** | 1 | 0.05 (-1.12,1.22) | 0.18 (-0.95,1.32) |
| **NaHCO₃** | -0.05 (-1.22,1.12) | 1 | 0.13 (-1.50,1.76) |
| **Cholecalciferol** | -0.18 (-1.32,0.95) | -0.13 (-1.76,1.50) | 1 |
| CI: confidence interval; PTH: parathyroid hormone; SMD: standardized mean difference. | | | |

| **Supplementary Table 13**. League table demonstrating the results of the network meta-analysis comparing the adverse events (OR, 95% CI). | | | | | |
| --- | --- | --- | --- | --- | --- |
| **Treatment** | **NaHCO₃** | **Protein** | **Control** | **Cholecalciferol** |  |
| **NaHCO₃** | 1 | 0.15 (-1.01,1.30) | 0.35 (-0.94,1.64)) | 0.85 (-0.66,2.35) |  |
| **Protein** | -0.15 (-1.30,1.01) | 1 | 0.20 (-0.39,0.79) | 0.70 (-0.27,1.67) |  |
| **Control** | -0.35 (-1.64,0.94) | -0.20 (-0.79,0.39) | 1 | 0.50 (-0.64,1.64) |  |
| **Cholecalciferol** | -0.85 (-2.35,0.66) | -0.70 (-1.67,0.27) | -0.50 (-1.64,0.6 | 1 |  |
| CI: confidence interval; OR: odds ratio. | | | | |  |

**
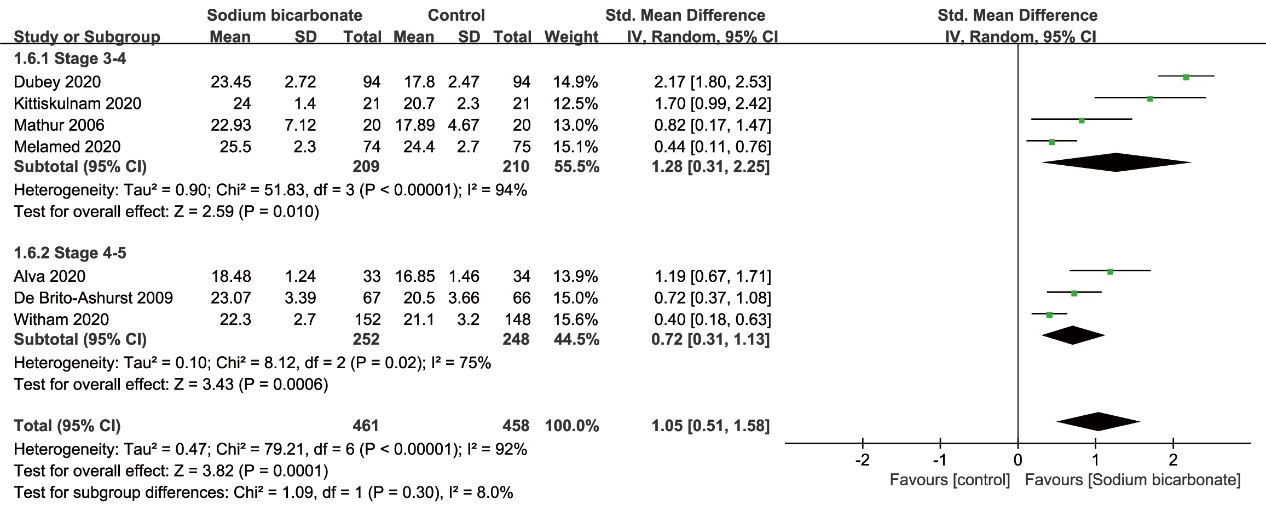
**

**Supplementary Figure 1**. Subgroup analysis showing the serum HCO₃⁻ levels in patients with different CKD stages following sodium bicarbonate intervention at 6 months.


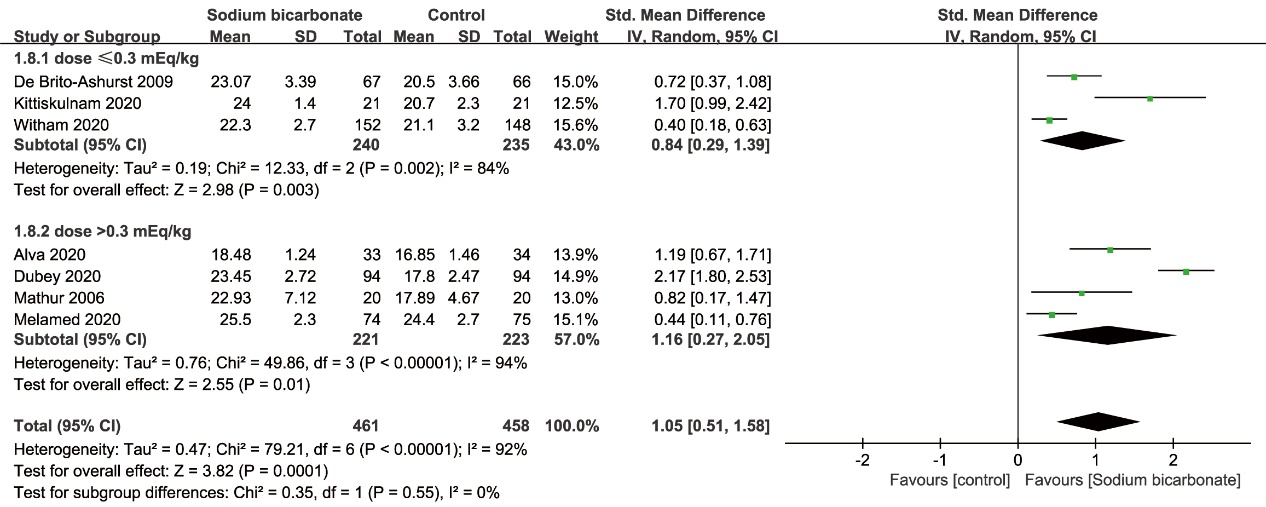


**Supplementary Figure 2**. Subgroup analysis showing the HCO₃⁻ levels in CKD patients receiving different doses of sodium bicarbonate at 6 months.


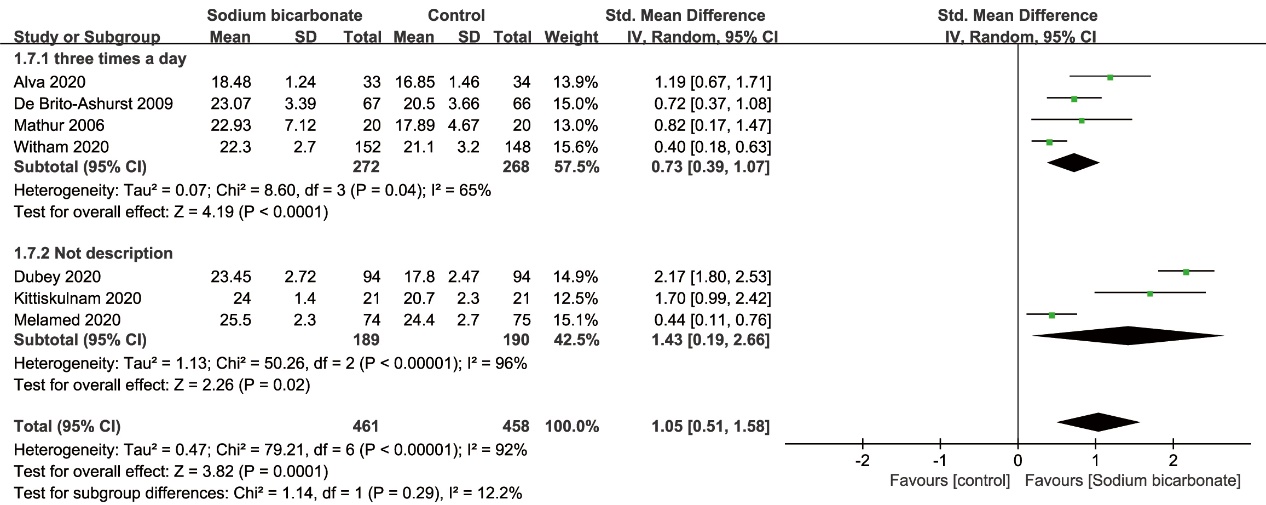


**Supplementary Figure 3**. Subgroup analysis of different intervention frequencies showing the serum HCO₃⁻ levels in CKD patients treated with sodium bicarbonate at 6 months.


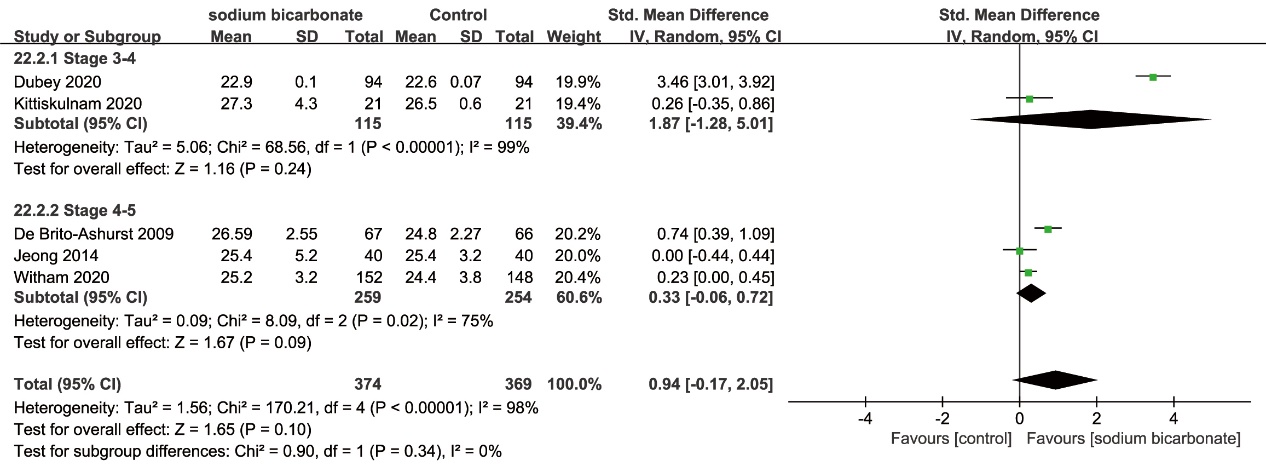


**Supplementary Figure 4**. Subgroup analysis showing the MAMC in patients with different CKD stages following sodium bicarbonate intervention at 12 months.


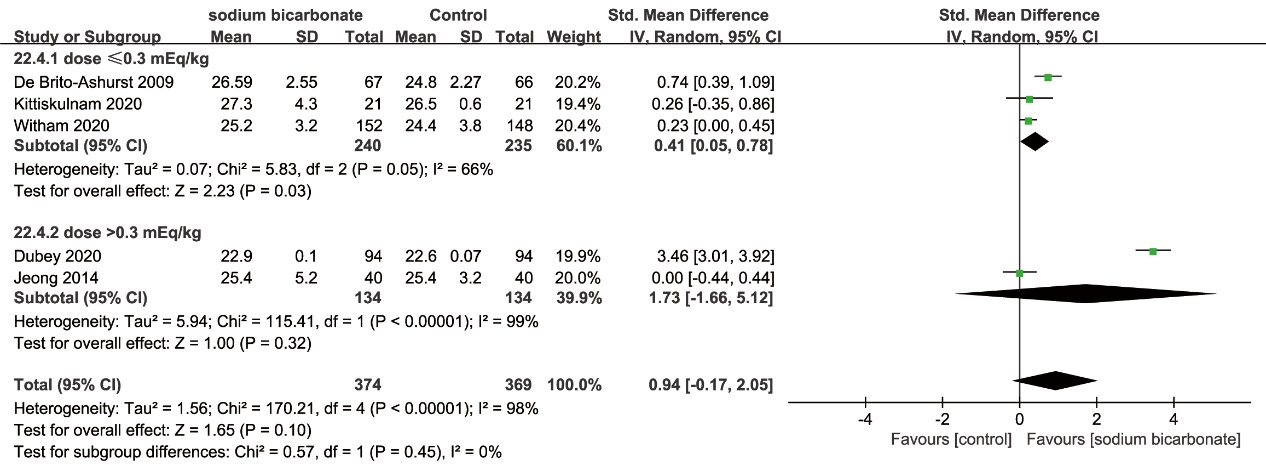


**Supplementary Figure 5**. Subgroup analysis showing the MAMC in CKD patients receiving different doses of sodium bicarbonate at 12 months.


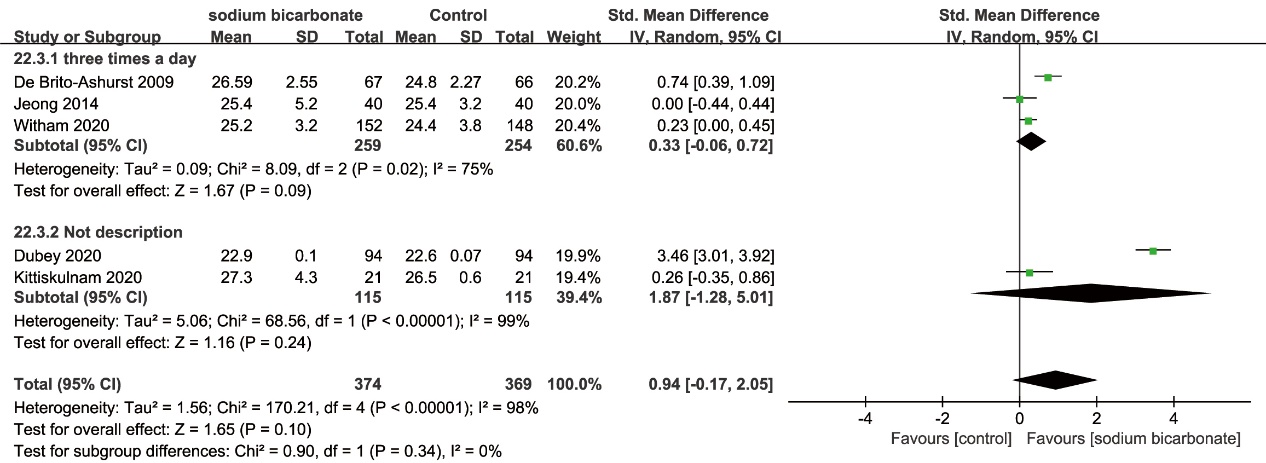


**Supplementary Figure 6**. Subgroup analysis of different intervention frequencies showing the MAMC in CKD patients treated with sodium bicarbonate at 12 months.


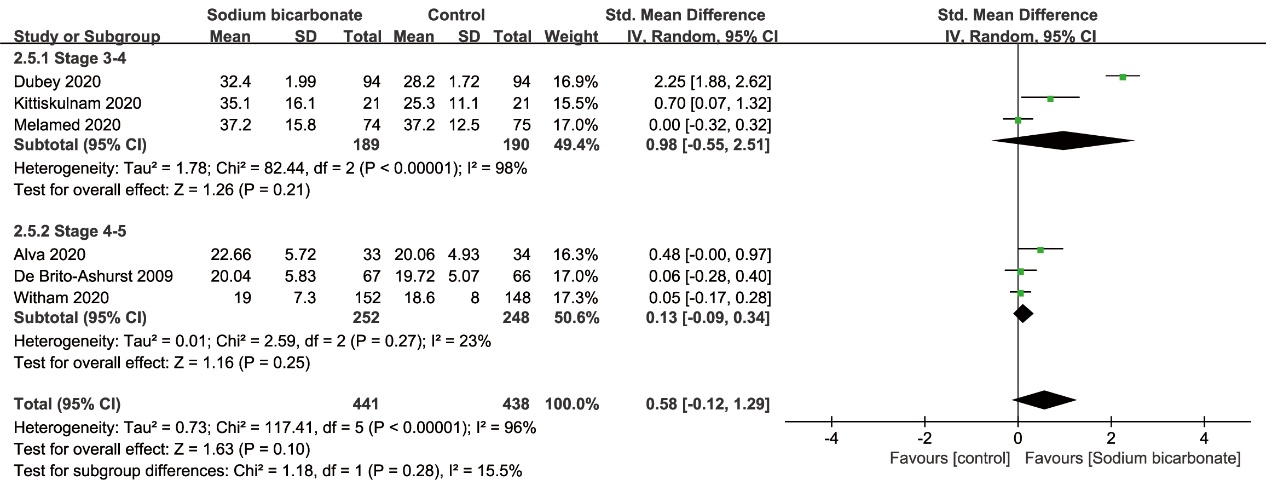


**Supplementary Figure 7**. Subgroup analysis showing the eGFR in patients with different CKD stages following sodium bicarbonate intervention at 6 months.


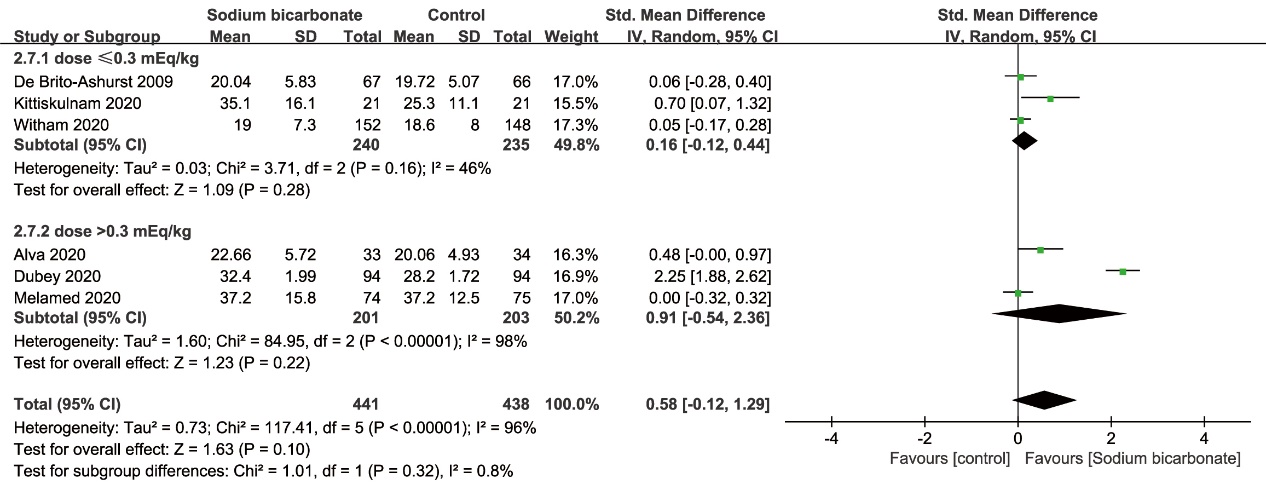


**Supplementary Figure 8**. Subgroup analysis showing the eGFR in CKD patients receiving different doses of sodium bicarbonate at 6 months.


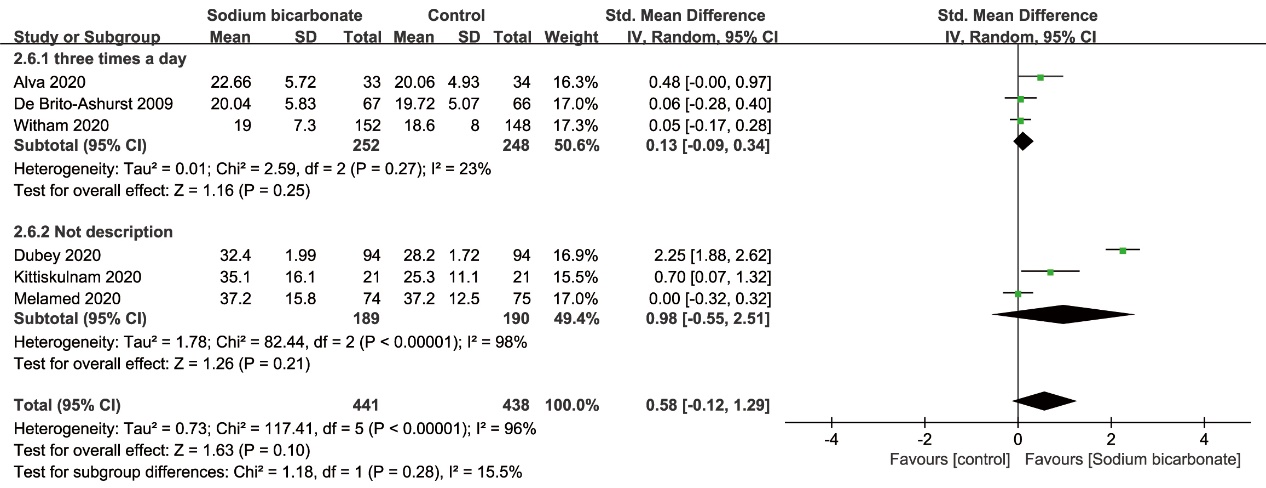


**Supplementary Figure 9**. Subgroup analysis of different intervention frequencies showing eGFR in CKD patients treated with sodium bicarbonate at 6 months.


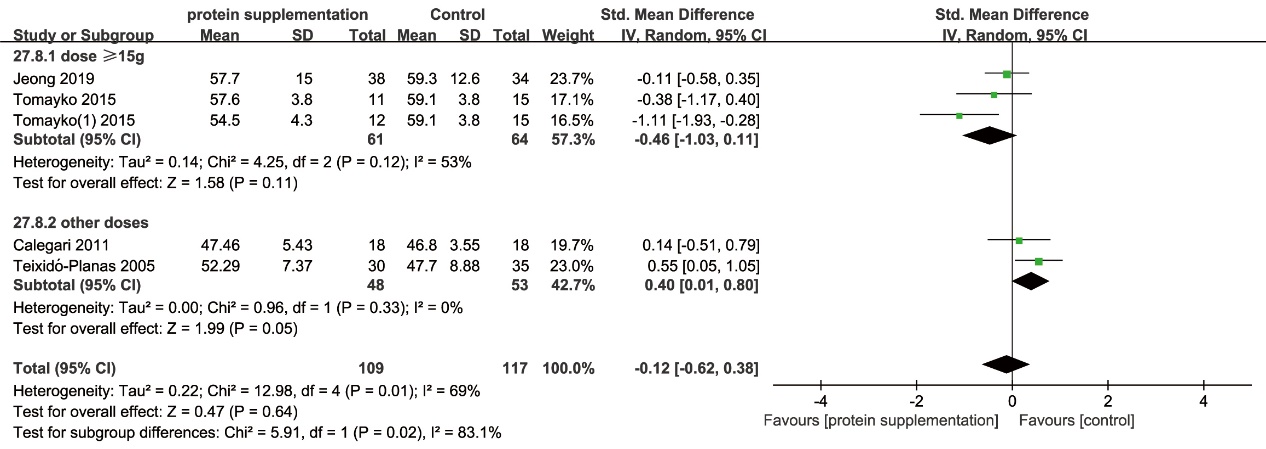


**Supplementary Figure 10**. Subgroup analysis showing the LBM in CKD patients receiving different doses of protein supplementation at 6 months.


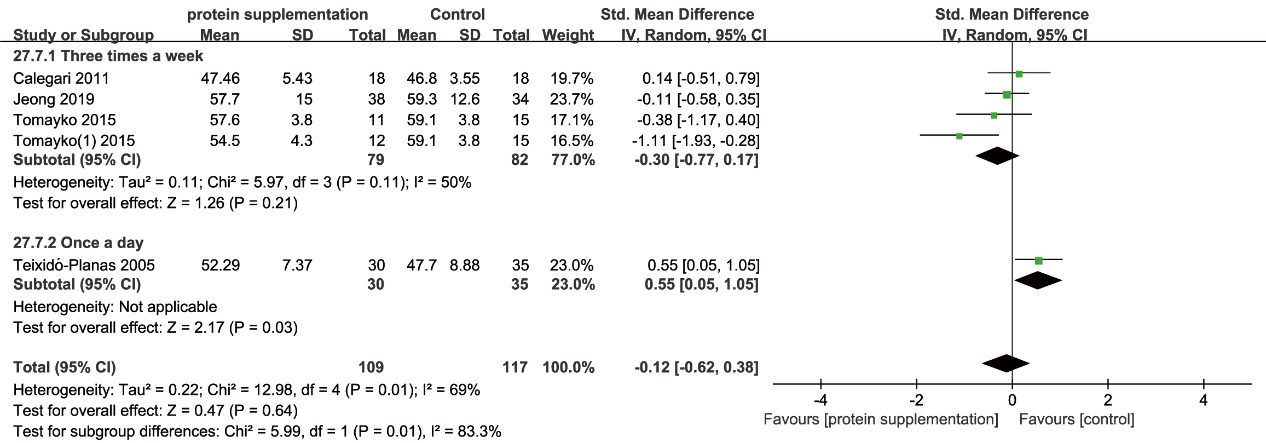


**Supplementary Figure 11**. Subgroup analysis of different intervention frequencies showing LBM in CKD patients treated with protein supplementation at 6 months.


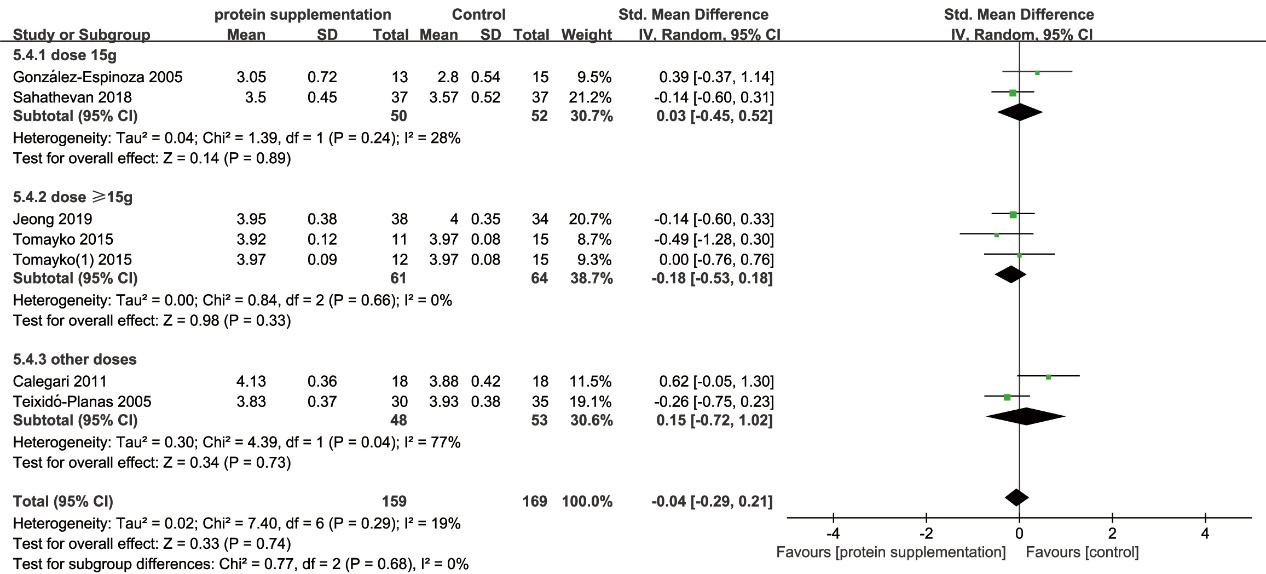


**Supplementary Figure 12**. Subgroup analysis showing the serum albumin in CKD patients receiving different doses of protein supplementation at 6 months.


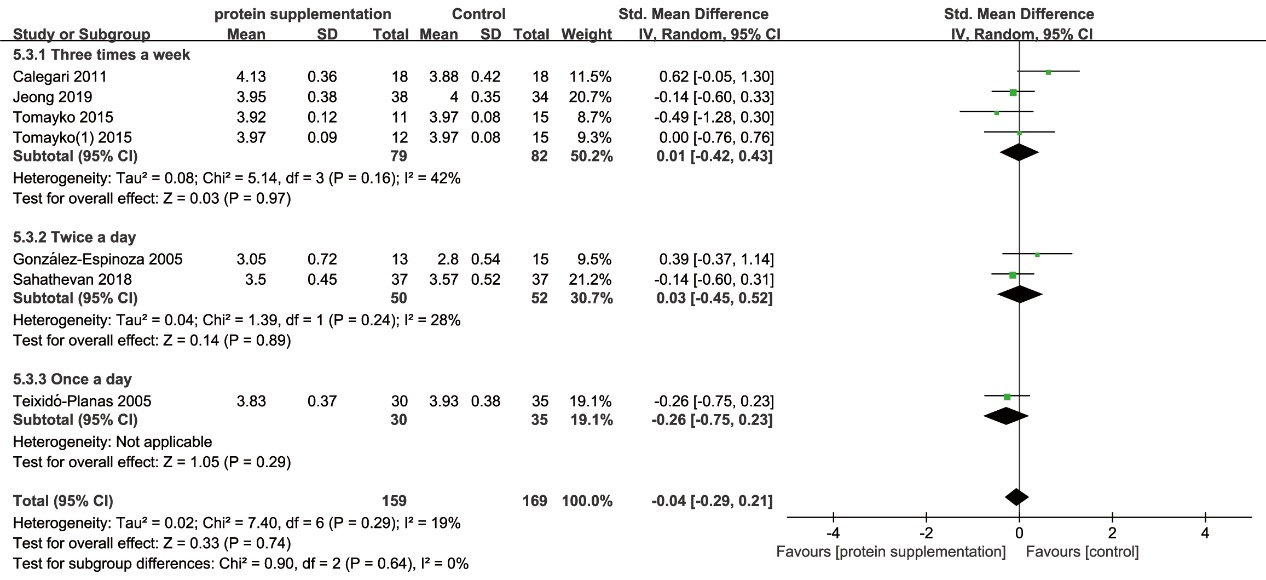


**Supplementary Figure 13**. Subgroup analysis of different intervention frequencies showing serum albumin in CKD patients treated with protein supplementation at 6 months.


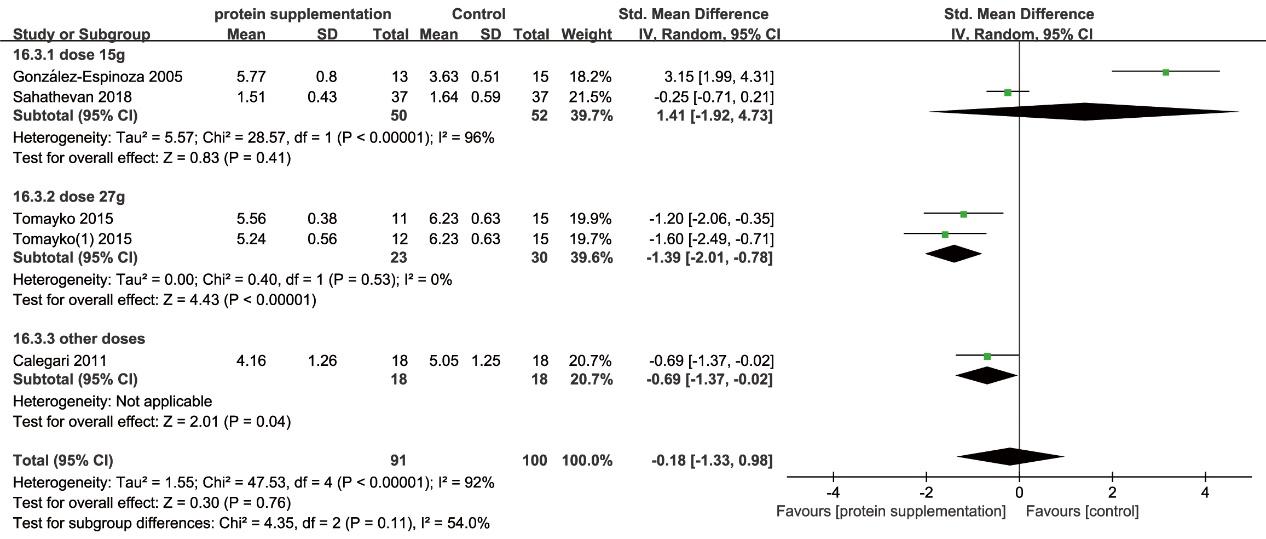


**Supplementary Figure 14**. Subgroup analysis showing the serum P in CKD patients receiving different doses of protein supplementation at 6 months.


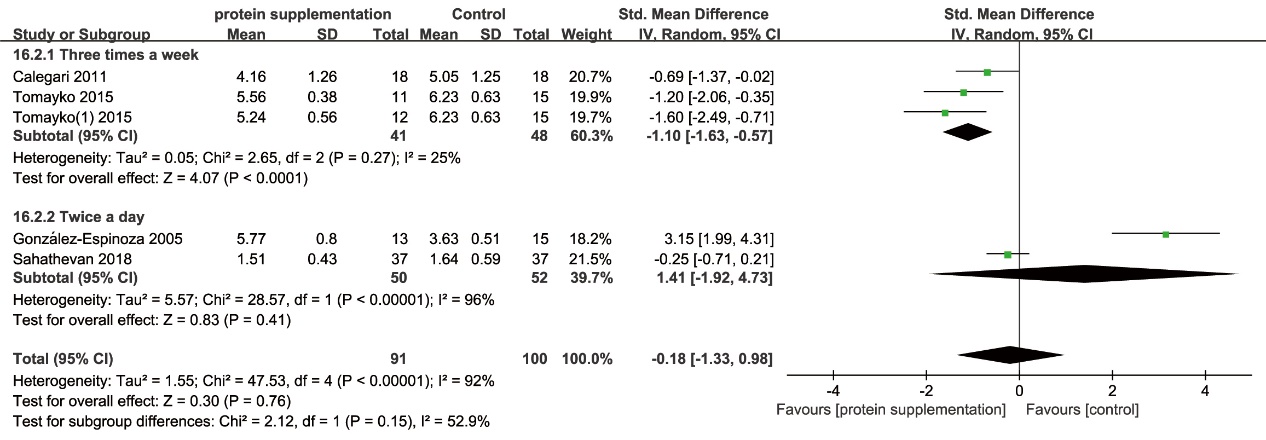


**Supplementary Figure 15**. Subgroup analysis of different intervention frequencies showing serum P in CKD patients treated with protein supplementation at 6 months.


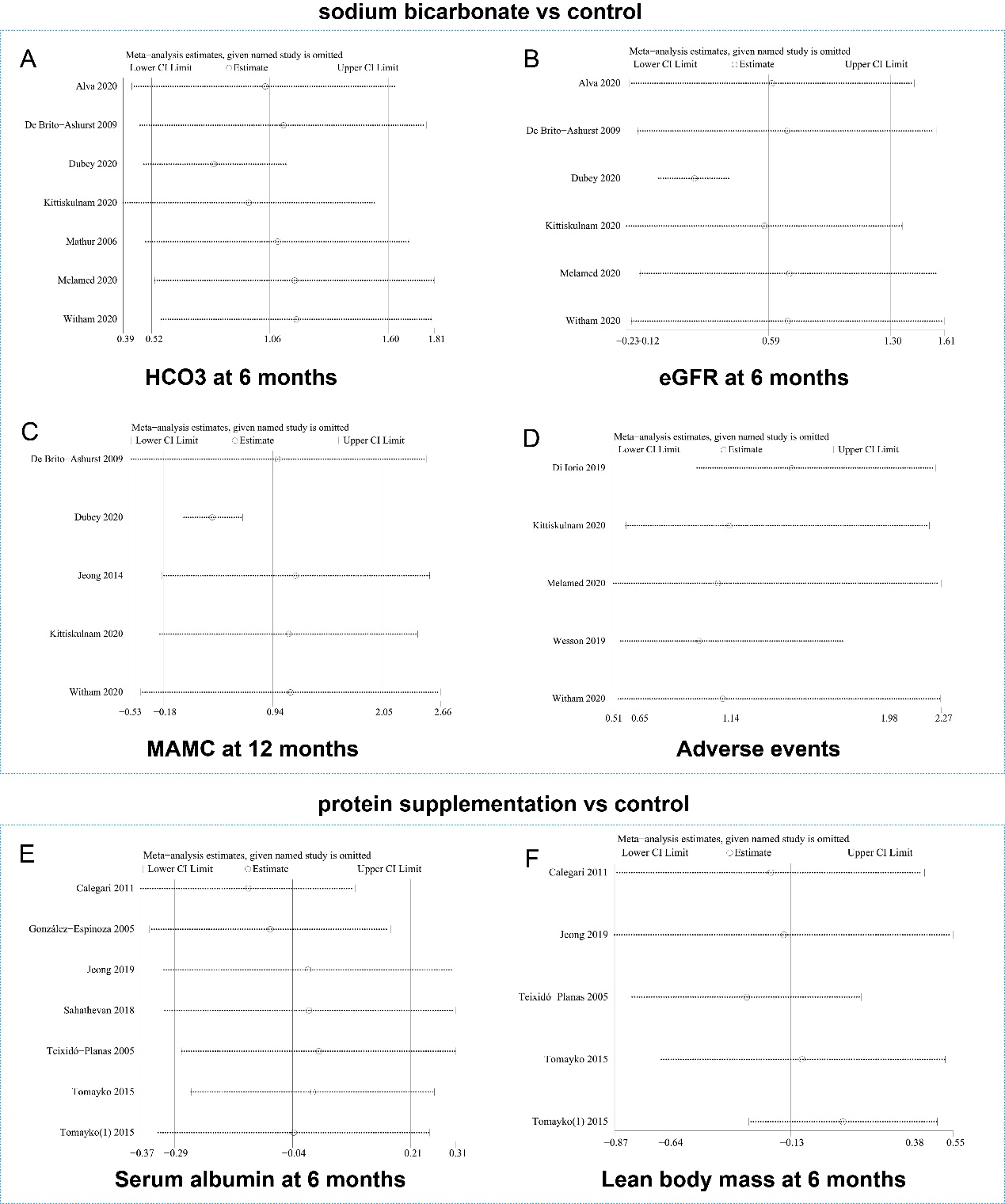


**Supplementary Figure 16**. Sensitivity analysis results of different key indicators. (A) HCO_3_ at 6 months. (B) sGFR at 6 months. (C) MAMC at 12 months. (D) The incidence of AEs. (E) Serum albumin at 6 months. (F) Lean body mass at 6 months.
